# Mitochondrial glycerol 3‐phosphate dehydrogenase promotes skeletal muscle regeneration

**DOI:** 10.15252/emmm.201809390

**Published:** 2018-11-02

**Authors:** Xiufei Liu, Hua Qu, Yi Zheng, Qian Liao, Linlin Zhang, Xiaoyu Liao, Xin Xiong, Yuren Wang, Rui Zhang, Hui Wang, Qiang Tong, Zhenqi Liu, Hui Dong, Gangyi Yang, Zhiming Zhu, Jing Xu, Hongting Zheng

**Affiliations:** ^1^ Translational Research Key Laboratory for Diabetes Department of Endocrinology Xinqiao Hospital Third Military Medical University Chongqing China; ^2^ Division of Endocrinology and Metabolism Department of Internal Medicine University of Virginia Health System Charlottesville VA USA; ^3^ Department of Gastroenterology Xinqiao Hospital Third Military Medical University Chongqing China; ^4^ Department of Endocrinology The Second Affiliated Hospital Chongqing Medical University Chongqing China; ^5^ Department of Hypertension and Endocrinology Daping Hospital Third Military Medical University Chongqing China

**Keywords:** diabetes, mGPDH, obesity, skeletal muscle regeneration, Metabolism, Musculoskeletal System, Regenerative Medicine

## Abstract

While adult mammalian skeletal muscle is stable due to its post‐mitotic nature, muscle regeneration is still essential throughout life for maintaining functional fitness. During certain diseases, such as the modern pandemics of obesity and diabetes, the regeneration process becomes impaired, which leads to the loss of muscle function and contributes to the global burden of these diseases. However, the underlying mechanisms of the impairment are not well defined. Here, we identify mGPDH as a critical regulator of skeletal muscle regeneration. Specifically, it regulates myogenic markers and myoblast differentiation by controlling mitochondrial biogenesis *via* CaMKKβ/AMPK. mGPDH^−/−^ attenuated skeletal muscle regeneration *in vitro* and *in vivo*, while mGPDH overexpression ameliorated dystrophic pathology in mdx mice. Moreover, in patients and animal models of obesity and diabetes, mGPDH expression in skeletal muscle was reduced, further suggesting a direct correlation between its abundance and muscular regeneration capability. Rescuing mGPDH expression in obese and diabetic mice led to a significant improvement in their muscle regeneration. Our study provides a potential therapeutic target for skeletal muscle regeneration impairment during obesity and diabetes.

## Introduction

Adult mammalian skeletal muscle is a stable tissue that is post‐mitotic; however, it actively undergoes regeneration following injury (Charge & Rudnicki, 2004). In certain diseases, such as the modern pandemics of obesity and diabetes, skeletal muscle regeneration becomes impaired, which leads to the loss of muscle function and contributes to the global burden of these diseases (Fu *et al*, 2016; Benoit *et al*, 2017). However, the mechanisms that underlie the regeneration impairment are poorly understood.

mGPDH is an integral component of the mitochondrial respiratory chain and functions as the rate‐limiting step in the glycerophosphate (GP) shuttle (Eto *et al*, 1999b). Due to a different structure and cell localization, the function and regulation of this enzyme are distinct from those of cytoplasmic glycerol 3‐phosphate dehydrogenase (cGPDH, also referred to as *GPD1*; Mracek *et al*, 2013). At the same time, despite the relatively simple structure of mGPDH, its functions remain largely unknown. Recently, mGPDH has been reported to be involved in hepatic glucose metabolism (Baur & Birnbaum, 2014; Madiraju *et al*, 2014). To gain a more complete understanding of mGPDH functions, we examined the role of mGPDH in skeletal muscle, which is a major insulin‐sensitive tissue that plays an essential role in glucose metabolism. Our results showed that although mGPDH is vital in regulating hepatic glucose metabolism, it did not significantly affect the glucose uptake and insulin signaling within skeletal muscle (differentiated C2C12 myoblasts). Interestingly, however, the mGPDH expression significantly increased over the course of C2C12 myocyte differentiation, with an expression profile similar to that of myogenic markers (myogenin and MyHC). These differentiation‐associated increases in mGPDH expression and activity were also clearly visible in mitochondrial fractions, which indicate that mGPDH might be involved in myogenic differentiation.

In the current study, we identify a novel characteristic of mGPDH in regulating myogenic differentiation and a potential therapeutic target for ameliorating muscle regeneration impairment and muscle pathology. In addition, the activation of the mGPDH/AMPK/mitochondrial biogenesis pathway of skeletal muscle might represent a new mechanism for treatment during obesity and diabetes.

## Results

### mGPDH regulates myoblast differentiation

**Figure 1:**
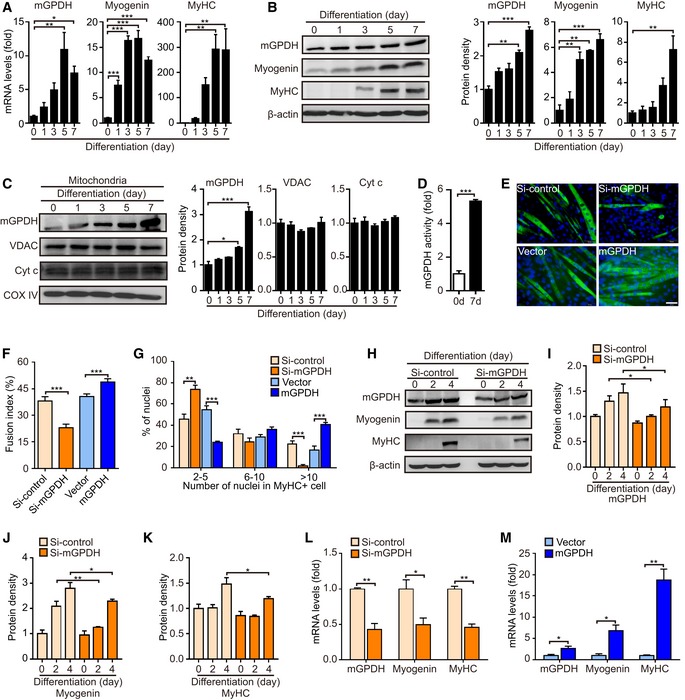
mGPDH regulates myoblast differentiation A, BqRT–PCR (A) and immunoblot (B) of mGPDH, myogenin, and myosin heavy chain (MyHC) levels during C2C12 myocyte differentiation. Quantification represents the levels of the indicated protein normalized to β‐actin.CImmunoblot of mGPDH, voltage‐dependent anion channel (VDAC), and cytochrome c (Cyt C) levels in mitochondrial lysate during C2C12 myocyte differentiation. Quantification represents the levels of the indicated protein normalized to COX IV.DActivity assay of mGPDH at days 0 and 7 after C2C12 myocyte differentiation.E–GRepresentative images of MyHC immunofluorescence (E) of C2C12 myocyte transfected with the siRNA or the overexpression plasmid for mGPDH; the fusion index (F) and the distribution of nuclei per myotube (G) were calculated at day 5 after differentiation.H–KImmunoblot of mGPDH, myogenin, and MyHC in C2C12 myocytes transfected with siRNA targeting mGPDH. Quantification (I–K) represents the levels of the indicated protein normalized to β‐actin at the indicated day after differentiation.L, MqRT–PCR analysis of mGPDH, myogenin, and MyHC in C2C12 myocytes transfected with the siRNA or the overexpression plasmid for mGPDH at day 4 after differentiation.Data information: Data are presented as the mean ± s.e.m. Scale bars represent 50 μm in panel (E). In panels (A–D) and (H–M), *n *=* *3; in panels (E–G), *n *=* *15. *P <* *0.05, **P <* *0.01, ***P <* *0.001. Unpaired *t*‐test was used for all analyses except in panel (G), where Kolmogorov–Smirnov test was used.Source data are available online for this figure.

**Figure EV1:**
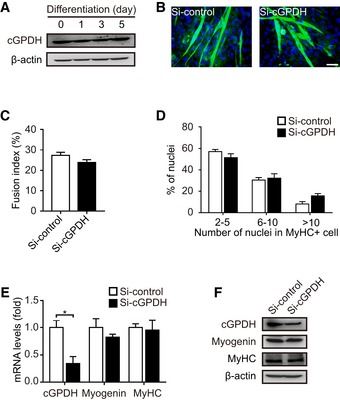
Effect of cGPDH on myoblast differentiation AcGPDH expression during C2C12 myocyte differentiation.B–DRepresentative images of MyHC immunofluorescence (B) of C2C12 myocytes transfected with siRNA targeting cGPDH; the fusion index (C) and the distribution of nuclei per myotube (D) were calculated.E, FqRT–PCR (E) and Western blot analysis (F) of myogenin and MyHC in C2C12 myocytes transfected with siRNA targeting cGPDH.Data information: Data are presented as the mean ± s.e.m. Scale bars represent 50 μm in panel (B). In panels (A–F), *n *=* *3. *P <* *0.05. Unpaired *t*‐test was used for all analyses except in panel (D), where the Kolmogorov–Smirnov test was used.

Our preliminary observations showed that mGPDH did not significantly influence glucose uptake or insulin signaling under both non‐insulin‐ and insulin‐treated conditions (Appendix Fig S1A and B), but its expression was augmented during the course of C2C12 myocyte differentiation (Fig 1A–D). To further explore the possibility of mGPDH involvement in myogenic differentiation, we regulated mGPDH expression by overexpression (plasmid pPR‐mGPDH) or inhibition (specific siRNA si‐mGPDH) in C2C12. Less cell fusion and multinuclear myotube formation events were observed in the si‐mGPDH group than those in the control, and striking increases in these events accompanied overexpression (Fig 1E–G). Furthermore, the protein expression of myogenin and MyHC was also reduced by si‐mGPDH during the course of differentiation (Fig 1H–K). Consistent changes in the corresponding mRNA levels following mGPDH expression perturbation were also identified (Fig 1L and M). These findings indicate that mGPDH is essential for myoblast differentiation. A major function of mGPDH is to form the GP shuttle with cGPDH. The expression of cGPDH was not significantly changed during the course of C2C12 myocyte differentiation, and the knockdown of cGPDH by siRNA did not show significant effects on C2C12 myocyte differentiation (Fig EV1A–F).

### mGPDH is essential in skeletal muscle regeneration

**Figure EV2:**
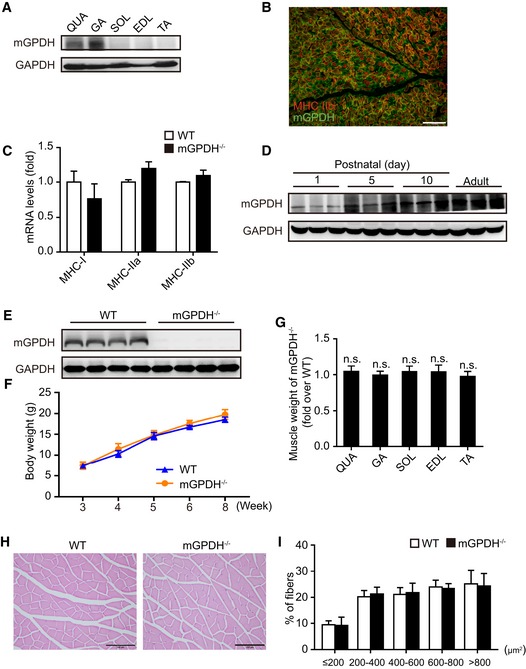
mGPDH is not essential to muscle development AImmunoblot of mGPDH in the quadriceps (QUA), gastrocnemius (GA), soleus (SOL), extensor digitorum longus (EDL), and tibialis anterior (TA) muscles of 8‐week‐old C57BL/6J mice.BImmunofluorescence showing localization of mGPDH with fiber type marker MHC IIb on cryosections from uninjured GA muscle of 8‐week‐old C57BL/6J mice.CqRT–PCR analyses of the indicated fiber type markers (MHC I, IIa, and IIb) in the uninjured GA muscles of 8‐week‐old WT and mGPDH^−/−^ mice.DImmunoblot of mGPDH in C57BL/6J mouse skeletal muscle at postnatal days 1, 5, and 10 and 8 weeks.EImmunoblot of mGPDH in the GA muscle of 8‐week WT and mGPDH^−/−^ mice.FBody weight of WT and mGPDH^−/−^ mice at the indicated week of age.GMuscle weight of the indicated 8‐week‐old mGPDH^−/−^ mice normalized to WT.H, IHematoxylin–eosin (H&E) staining (H) and average myofiber cross‐sectional area (CSA) (I) in the GA muscle of 8‐week‐old WT and mGPDH^−/−^ mice.Data information: Data are presented as the mean ± s.e.m. Scale bars represent 200 μm in panel (B) and 100 μm in panel (H). In panels (A and D), *n *=* *3 mice per group; in panels (B and C), *n *=* *6 mice per group; in panels (E–I), *n *=* *4 mice per group; in panels (H and I), three sections were obtained per mouse. n.s., not significant. Unpaired *t*‐test was used for all analyses except in panel (I), where the Kolmogorov–Smirnov test was used.

Myoblast differentiation occurs during muscle development and also during adulthood for muscle mass maintenance and muscle regeneration (Charge & Rudnicki, 2004). Here, we aim to identify the role of mGPDH in both stages. First, we examined the mGPDH distribution among different skeletal muscles and found that it was abundantly expressed in the gastrocnemius (GA) and quadriceps femoris (QUA), particularly in the GA (Fig EV2A); it seems that mGPDH does not match with the muscle fiber type. To further observe this issue, we costained MHC IIb (the most abundant fiber type in GA muscle) with mGPDH. The results showed the fibers were stained as three colors (Fig EV2B), which indicates that mGPDH did not match with the fiber type in GA muscle. Moreover, the expressions of MHC isoforms (MHC I, IIa, and IIb) were not significantly changed in mGPDH‐depleted skeletal muscle (Fig EV2C). During muscle development, the mGPDH expression increased after birth, but only for the first few postnatal days (Fig EV2D). In the mGPDH knockout (mGPDH^−/−^) mice (Fig EV2E), there were no significant differences in the body and muscle weight compared with the wild‐type (WT) mice during development (Fig EV2F and G). Histological analysis also showed no differences in the muscle appearance or myofiber size between these two genotypes (Fig EV2H and I), which suggests mGPDH is not essential for muscle development.

**Figure 2:**
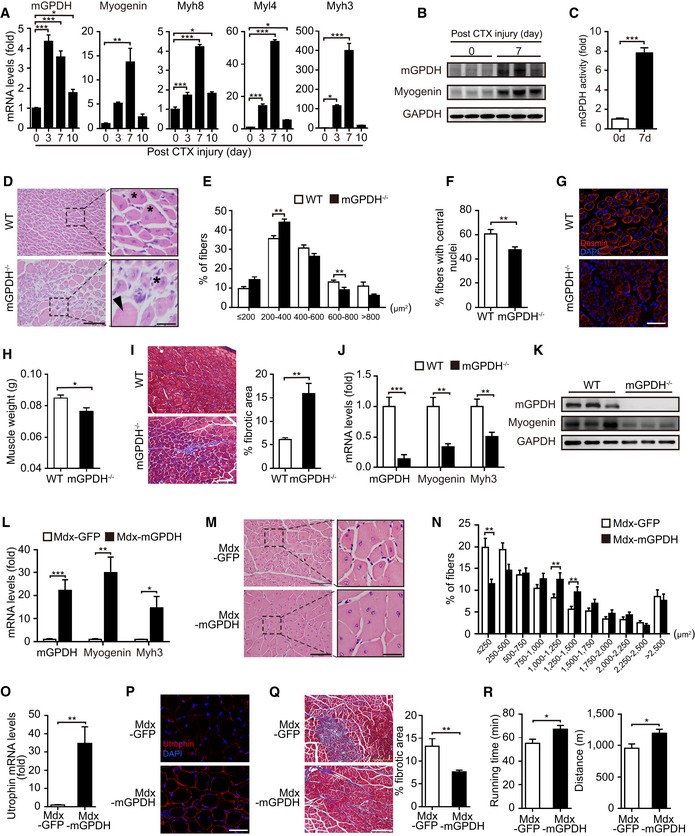
mGPDH is essential to skeletal muscle regeneration A, BqRT–PCR (A) and immunoblot (B) of mGPDH, myogenin, and developmental myosin heavy chain (myh8, myl4, and myh3) in gastrocnemius (GA) muscle from C57BL/6J mice at the indicated day after CTX intramuscular injection.CActivity assay of mGPDH in GA muscle from C57BL/6J mice at days 0 and 7 after CTX injection.D–GRepresentative images of the H&E staining (arrowhead, necrotic myofibers; asterisks, regenerating fibers) (D), distribution of the fiber cross‐sectional area (CSA) (E), percentage of myofibers with central nuclei (F), and immunofluorescence staining of desmin (green) (G) in GA muscle from WT and mGPDH^−/−^ mice at day 7 post‐CTX injection.H, IMuscle weight (H) and trichrome staining (I) in GA muscle from WT and mGPDH^−/−^ mice at day 14 post‐CTX injection. Quantification represents the fibrotic areas.J, KqRT–PCR (J) and immunoblot (K) for mGPDH, myogenin, and myh3 in GA muscle from WT and mGPDH^−/−^ mice at day 7 post‐CTX injection.L–QqRT–PCR for mGPDH, myogenin, and myh3 (L), H&E staining (M), distribution of the fibers CSA (N), qRT–PCR (O), and immunofluorescence staining (P) for utrophin and trichrome staining (Q) in GA muscle from mdx mice 4 weeks after AAV‐mGPDH intramuscular injection.RExercise capacity of mdx mice 6 weeks after AAV‐mGPDH tail vein injection.Data information: Data are presented as the mean ± s.e.m. Scale bars represent 100 μm (25 μm for magnification insets) in panels (D, I, M, and Q) and 50 μm in panels (G, P). In panels (A–C), *n *=* *3; in panels (D–R), *n *=* *6 mice per group; in panels (D–F, M, and N), three sections were obtained per mouse. *P <* *0.05, **P <* *0.01, ***P <* *0.001. Unpaired *t*‐test was used for all analyses except in panels (E, N), where the Kolmogorov–Smirnov test was used.Source data are available online for this figure.

Thus, we subsequently assessed the role of mGPDH in muscle regeneration post‐injury. Both the mGPDH expression and activity were increased in GA muscle after cardiotoxin (CTX) injury and paralleled the changes of myogenic markers and developmental myosin heavy chain (Fig 2A–C), which is consistent with our observation *in vitro* (Fig 1A–D). In addition, compared with the basal expression of mGPDH in normal fibers with peripheral nuclei, the injury‐induced higher expression of mGPDH was mainly localized in regenerating fibers with central nuclei (Appendix Fig S2), which indicates the injury‐induced mGPDH expression predominately presented in newly formed myofibers. Although both the mGPDH^−/−^ and WT mice exhibited extensive muscle damage at day 3 post‐injury, the mGPDH^−/−^ mice showed a delay in the disappearance of necrotic fibers and inflammatory cells and had fewer and more unevenly distributed newly formed myofibers with multiple centrally located nuclei at day 7 (Fig 2D–F). The immunofluorescence of desmin, an intermediate filament protein in newly generated myofibers (Liu *et al*, 2012), further confirmed the impaired muscle regeneration in mGPDH^−/−^ mice (Fig 2G). At day 14, the muscle weight was decreased (Fig 2H), while the collagen deposition was increased (Fig 2I) in the mGPDH^−/−^ mice. These results suggested that mGPDH loss attenuates muscle regeneration. At the same time, expressions of the satellite cell marker paired box protein 7 (PAX7; Zhang *et al*, 2016; Bi *et al*, 2017) and the satellite cell activation marker myoblast determination protein (MyoD; Zhang *et al*, 2016; Bi *et al*, 2017) were not different between the mGPDH^−/−^ and WT mice (Appendix Fig S3A–F), which suggests that mGPDH has no significant effects on myoblast quantity and activation. However, the differentiation markers myogenin and myh3 (Park *et al*, 2016) were reduced in the mGPDH^−/−^ mice (Fig 2J and K), which is consistent with our *in vitro* data and indicates that mGPDH deletion inhibits skeletal muscle regeneration by diminishing myoblast differentiation.

Next, we activated mGPDH *via* AAV in mdx mice, which represent a model of Duchenne muscular dystrophy, in which there is a persistent damage and loss of myofibers induced by the *Dmd* gene mutation (Barton *et al*, 2002; Duddy *et al*, 2015; Novak *et al*, 2017). The basal expression levels of mGPDH and myogenin were increased in the mdx mice compared to the normal mice, which indicated an activated regeneration process that was insufficient to compensate (Appendix Fig S4A and B). The overexpression of mGPDH via intramuscular injection of AAV into the GA muscle induced a further increase in myogenin and myh3 expression (Fig 2L); in line with this finding, the number of small regenerating fibers and the variability in the myofiber size were decreased (Fig 2M), and the distribution of the cross‐sectional area (CSA) shifted to the right (Fig 2N). Moreover, the mRNA and protein levels of utrophin, an indicator of regeneration in mdx mice (Durko *et al*, 2010), were increased (Fig 2O and P), while muscle fibrosis decreased (Fig 2Q). Furthermore, systematically up‐regulating mGPDH via tail vein injection of AAV improved the exercise capacity of the mdx mice (Appendix Fig S5 and Fig 2R). Taken together, these *in vivo* data of mGPDH deletion and overexpression suggest that mGPDH plays a pivotal role in regulating myoblast differentiation and muscle regeneration.

### mGPDH effects occur *via* the CaMKKβ/AMPK control of mitochondrial biogenesis

**Figure 3:**
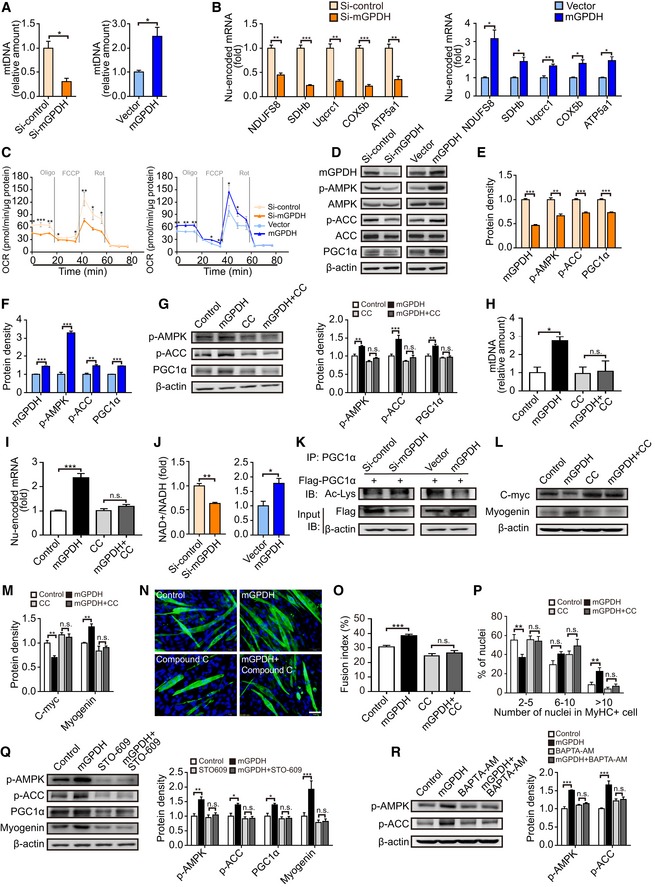
mGPDH effect occurs via the CaMKKβ/AMPK control of mitochondrial biogenesis A–FMitochondrial DNA (A), nuclear‐encoded OXPHOS genes (B), respirometry analysis (C), and immunoblots of mGPDH, phospho‐Thr172 AMPK (p‐AMPK), total AMPK (AMPK), phospho‐Ser79‐ACC (p‐ACC), total ACC and PGC1α, and corresponding quantifications represent mGPDH, p‐AMPK, p‐ACC, and PGC1α protein levels (D–F) in C2C12 myocytes transfected with siRNA or plasmid for mGPDH 24 h after differentiation.G–IImmunoblots of p‐AMPK, p‐ACC, and PGC1α and corresponding quantifications represent p‐AMPK, p‐ACC, and PGC1α protein levels (G), mitochondrial DNA (H), and nuclear‐encoded OXPHOS genes combined by *NDUFS8*,* SDHb*,* Uqcrc1*,* COX5b*, and *ATP5a1* (I) in C2C12 myocytes transfected by mGPDH plasmid with the AMPK inhibitor compound C (CC) 24 h after differentiation.J, KNAD^+^/NADH ratio (J) and immunoprecipitation analysis for PGC1α acetyl‐lysine (Ac‐Lys) level (K) in C2C12 myocytes transfected with siRNA or plasmid for mGPDH 24 h after differentiation.L–PImmunoblot of c‐myc and myogenin (L) and corresponding quantifications represent c‐myc and myogenin protein levels (M), representative images of MyHC immunofluorescence (N), fusion index (O), and the distribution of nuclei per myotube (P) in C2C12 myocytes transfected with mGPDH plasmid with the AMPK inhibitor CC at 24 h (L, M) or 72 h (N–P) after differentiation.QImmunoblots of p‐AMPK, p‐ACC, PGC1α, and myogenin in C2C12 myocytes transfected with mGPDH plasmid with the CaMKKβ inhibitor STO‐609 at 24 h after differentiation. Quantifications represent p‐AMPK, p‐ACC, PGC1α, and myogenin protein levels.RImmunoblots of p‐AMPK and p‐ACC in C2C12 myocytes transfected with mGPDH plasmid with the Ca^2+^ chelator BAPTA‐AM at 24 h after differentiation. Quantifications represent p‐AMPK and p‐ACC protein levels.Data information: Data are presented as the mean ± s.e.m. Scale bars represent 50 μm in panel (N). In panels (A, B, D–M, Q, and R), *n *=* *3; in panel (C), *n *=* *10; in panels (N–P), *n *=* *15. *P <* *0.05, **P <* *0.01, ***P <* *0.001, n.s.: not significant. Unpaired *t*‐test was used in panels (A–C, E, F, and J); one‐way ANOVA with Tukey's comparison test was used in panels (G–I, M, O, Q, and R); and the Kolmogorov–Smirnov test was used in panel (P).Source data are available online for this figure.

To gain further insights into the underlying molecular mechanisms, we subsequently assessed a number of the common factors related to myoblast differentiation, such as the cell cycle, apoptosis, autophagy, insulin‐like growth factor‐1 (IGF‐1), and mitochondrial biogenesis (Musaro *et al*, 2001; Kim *et al*, 2010; Hochreiter‐Hufford *et al*, 2013; Zhang *et al*, 2014; Garcia‐Prat *et al*, 2016). mGPDH had no significant effects on the cell cycle, apoptosis, autophagy, and IGF‐1 receptor expression (Appendix Fig S6A–D), but obviously changed the mitochondrial content of C2C12 myocytes (Fig 3A). Moreover, it regulated the expression of nuclear‐encoded oxidative phosphorylation (OXPHOS) subunits (*NDUFS8, SDHb, Uqcrc1, COX5,* and *ATP5a*; Fig 3B), despite no substantial impact on the mitochondrial genomes (*ND1, Cytb, COX1,* and *ATP6*; Appendix Fig S7A and B). The mitochondrial respiration rate further confirmed these links to mitochondrial biogenesis (Fig 3C). AMP‐activated protein kinase (AMPK) is a key regulator of nuclear‐encoded OXPHOS subunits and mitochondrial function (Xiao *et al*, 2011; Lin *et al*, 2012; Gomes *et al*, 2013; Mottillo *et al*, 2016), and it has also been reported to influence myoblast differentiation (Mounier *et al*, 2013). Therefore, we assessed whether AMPK is involved in mGPDH effects. As shown in Fig 3D, mGPDH expression significantly activated AMPK and its downstream acetyl‐CoA carboxylase (ACC), as well as the mitochondrial biogenesis marker peroxisome proliferator‐activated receptor‐γ coactivator‐1α (PGC1α; Fig 3D–F). Strikingly, the activated mitochondrial biogenesis caused by mGPDH overexpression, including increased PGC1α, mitochondrial content, and nuclear‐encoded OXPHOS, was abrogated when the AMPK inhibitor compound C was applied (Fig 3G–I), which indicates that the effects of mGPDH on nuclear‐encoded OXPHOS subunits and mitochondrial biogenesis are AMPK‐dependent. Previous studies have reported that to a large extent, AMPK regulated mitochondrial biogenesis mainly through the modulation of PGC1α activity by the NAD^+^/NADH ratio (Iwabu *et al*, 2010; Meng *et al*, 2013; Woldt *et al*, 2013). Our results showed that mGPDH loss‐ and gain‐of‐function manipulations affected the NAD^+^/NADH ratio (Fig 3J). Moreover, PGC1α acetylation was altered when mGPDH expression changed (Fig 3K). Mitochondrial biogenesis is critical for skeletal muscle differentiation. It activates myoblast differentiation markers by suppressing c‐myc expression, which represses myoblast differentiation through direct binding to the promoters or enhancers of myogenin (Miner & Wold, 1991; Seyer *et al*, 2010; Ravel‐Chapuis *et al*, 2014). In our observation, mGPDH overexpression repressed c‐myc expression and increased myogenin expression and myoblast differentiation, and these effects were abolished by the AMPK inhibitor compound C (Fig 3L–P).

AMPK activity is dependent on the phosphorylation of AMPKα (Thr172) by AMPK kinases (AMPKKs; Iwabu *et al*, 2010). To further clarify the link between mGPDH and AMPK, two major AMPKKs, liver kinase B1 (LKB1) and Ca2^+^/calmodulin‐dependent protein kinase kinase (CaMKK) β (Hawley *et al*, 2005; Kahn *et al*, 2005; Woods *et al*, 2005), were assessed. LKB1 siRNA did not hinder the regulation of mGPDH on AMPK (Appendix Fig S8), in contrast to the CaMKKβ‐specific inhibitor STO‐609 (Fig 3Q). We subsequently explored how mGPDH regulates cytoplasmic CaMKKβ. Previous studies have demonstrated that intracellular Ca^2+^ plays an important role in the crosstalk between mitochondria and the cytoplasm (Ganitkevich, 2003; Fieni *et al*, 2014; Stefani *et al*, 2016), and Ca^2+^ has also been reported to serve as the initiating factor during the cascade involving CaMKKβ‐activated AMPK (Iwabu *et al*, 2010; Marcelo *et al*, 2016). Therefore, we used a cell membrane‐permeable Ca^2+^ chelator, BAPTA‐AM, to eliminate intracellular free Ca^2+^ and found that mGPDH‐induced AMPK activation was abolished (Fig 3R). Together, these data suggest that the regulation of myoblast differentiation by mGPDH occurs *via* CaMKKβ/AMPK control of mitochondrial biogenesis.

### Rescuing mGPDH deficiency improves skeletal muscle regeneration during obesity and diabetes

**Figure EV3:**
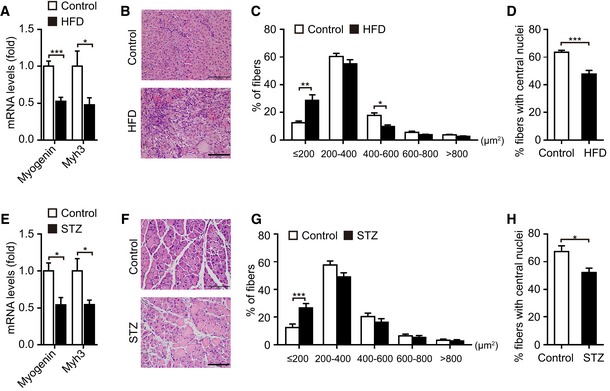
Skeletal muscle regeneration is impaired in HFD‐fed mice and STZ‐injected mice A–DGastrocnemius (GA) muscles were obtained from HFD‐fed mice at day 7 post‐CTX injury. Quantification of myogenin and myh3 by qRT–PCR (A), representative images of H&E staining (B), distribution of the CSA (C), and percentage of myofibers with central nuclei (D).E–HGA muscles were obtained from STZ‐treated mice 4 weeks after STZ injection and at day 7 post‐CTX injury. Quantification of myogenin and myh3 by qRT–PCR (E), representative images of H&E staining (F), distribution of the fibers CSA (G), and percentage of myofibers with central nuclei (H).Data information: Data are presented as the mean ± s.e.m. Scale bars represent 100 μm in panels (B and F). *n *=* *6 mice per group. *P <* *0.05, **P <* *0.01, ***P <* *0.001. Unpaired *t*‐test was used for all analyses except in panels (C and G), where the Kolmogorov–Smirnov test was used.

**Figure 4:**
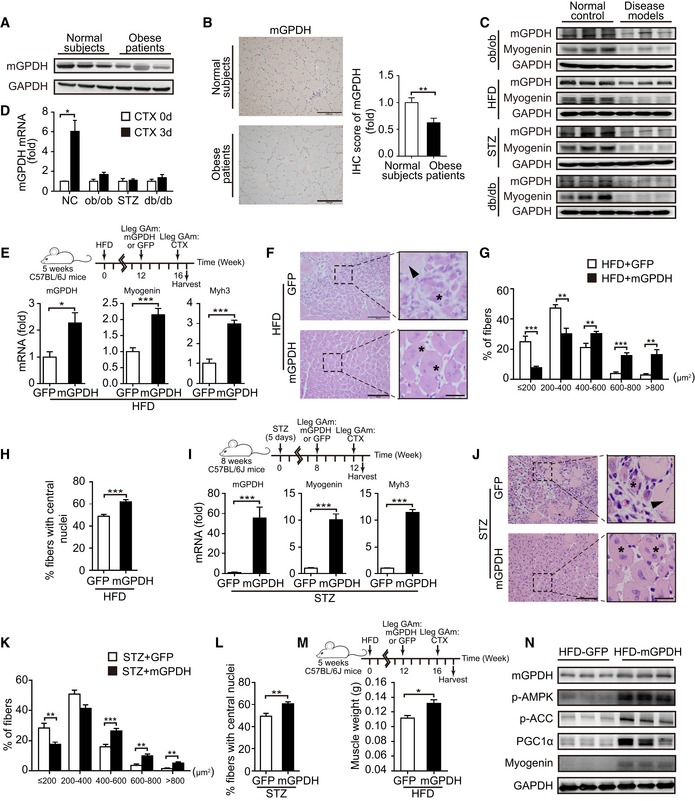
Rescuing mGPDH deficiency improves skeletal muscle regeneration during obesity and diabetes A–CImmunoblot (A, C) and IHC (B) of mGPDH and myogenin in GA muscles from obese patients (A, B) and the indicated mice (C).DqRT–PCR of mGPDH in GA muscle of the indicated mice at days 0 and 3 after CTX intramuscular injection.E–HExperimental setup (E, upper panel); qRT–PCR of mGPDH, myogenin, and myh3 (E, bottom panel); H&E staining (arrowhead, necrotic myofibers; asterisks, regenerating fibers) (F); distribution of the fiber CSA (G); and percentage of myofibers with central nuclei (H) in GA muscle from AAV‐mGPDH‐treated HFD‐fed mice at day 7 after CTX intramuscular injection.I–MExperimental setup (I and M, upper panels); qRT–PCR of mGPDH, myogenin, and myh3 (I, bottom panel); H&E staining (arrowhead, necrotic myofibers; asterisks, regenerating fibers) (J); distribution of the fibers CSA (K); percentage of myofibers with central nuclei (L); and muscle weight (M, bottom panel) in GA muscle from AAV‐mGPDH‐treated STZ‐treated mice at days 7 (I–L) and 14 (M) after CTX intramuscular injection.NImmunoblots of mGPDH, p‐AMPK, p‐ACC, PGC1α, and myogenin for the experiment described in (E).Data information: Data are presented as the mean ± s.e.m. Scale bars represent 200 μm in panel (B) and 100 μm (25 μm for magnification insets) in panels (F, J). In panels (A, B), obese patients (*n *=* *11) and normal subjects (*n *=* *18); in panels (C, D), *n *=* *3 mice per group; in panels (E–L and N), *n *=* *6 mice per group; in panel (M), *n *=* *4 mice per group; in panels (B, F–H, and J–L), three sections were obtained per mouse. *P <* *0.05, **P <* *0.01, ***P <* *0.001. Unpaired *t*‐test was used in panels (D, E, H, I, L, and M); the Wilcoxon test was used in panel (B); and the Kolmogorov–Smirnov test was used in panels (G, K).Source data are available online for this figure.

**Figure EV4:**
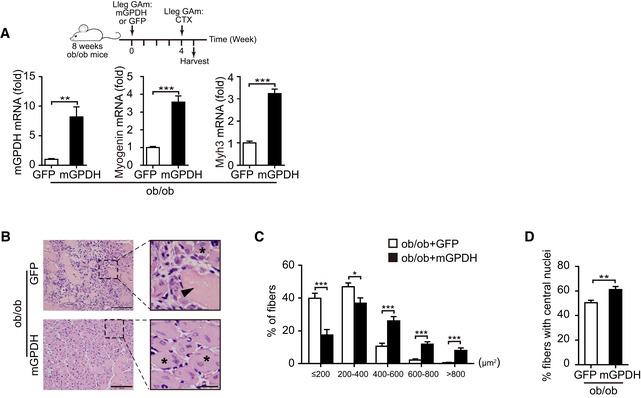
mGPDH activation improves skeletal muscle regeneration in *ob*/*ob* mice A–DExperimental setup (A, upper panel) and qRT–PCR of mGPDH, myogenin, and myh3 gene expressions (A, bottom panel), H&E staining (arrowhead, necrotic myofibers; asterisks, regenerating fibers) (B), distribution of CSA (C), and percentage of fibers with central nuclei (D) in GA muscle from AAV‐mGPDH‐treated *ob*/*ob* mice at day 7 post‐CTX.Data information: Data are presented as the mean ± s.e.m. Scale bars represent 100 μm (25 μm for magnification insets) in panel (B). In panels (A–D), *n *=* *6 mice per group; in panels (B–D), three sections were obtained per mouse. *P <* *0.05, **P <* *0.01, ***P <* *0.001. Unpaired *t*‐test was used for all analyses except in panel (C), where the Kolmogorov–Smirnov test was used.

**Figure EV5:**
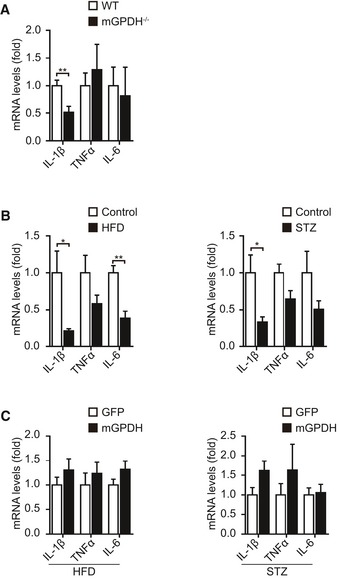
Effect of mGPDH on inflammatory signaling A–CQuantification of the indicated inflammatory cytokines by qRT–PCR in GA muscles of mGPDH^−/−^ mice (A), HFD‐fed and STZ‐treated mice (B), and HFD‐fed and STZ‐treated mice intramuscularly injected with AAV‐mGPDH (C) at day 7 post‐CTX injury.Data information: Data are presented as the mean ± s.e.m. *n *=* *6 mice per group. *P <* *0.05, **P <* *0.01. Unpaired *t*‐test was used for all panels.

Based on the observed effects of mGPDH on myoblast differentiation and muscle regeneration, we then explored its role under pathological conditions. Skeletal muscle regeneration was impaired in obese and diabetic mice (Fig EV3A–H), which was consistent with previous observations (Aragno *et al*, 2004; Fu *et al*, 2016). In patients, approximately 60% of obese patients have a low muscle mass, decreased muscle strength, and poor physical function (Chiu *et al*, 2016; Fu *et al*, 2016; Lee *et al*, 2016; Benoit *et al*, 2017). Our results showed a lower mGPDH expression in the GA muscle of obese patients compared with healthy subjects (ChiCTR‐ROC‐17010719; Fig 4A and B, and Appendix Table S1). These results were replicated in obese (*ob*/*ob* and HFD), diabetic (STZ), and diabetic–obese (*db*/*db*) mice, accompanied by myogenin reduction (Fig 4C). mGPDH activation during the regeneration process was also defective in these models (Fig 4D). In contrast, the cGPDH expressions were not notably altered in obese (HFD), diabetic (STZ), and mdx mice. Moreover, the expressions did not change during the regeneration process post‐CTX injection (Appendix Fig S9). Thus, we observed the effects of rescuing mGPDH expression on muscle regeneration during obesity and diabetes. By day 7 post‐CTX injury, the AAV‐mGPDH‐treated group had higher expressions of myogenin and myh3 in the GA muscle from both the obese (HFD and *ob*/*ob*) and diabetic (STZ) mice (Figs 4E and I, and EV4A). Histological analysis showed enhanced muscle regeneration, evidenced by a reduction of necrotic fibers, a right‐shifted CSA distribution, and an increased number of newly formed myofibers with central myonuclei across these animal models (Figs 4F–H and J–L, and EV4B–D). The AAV‐mGPDH mice also exhibited a higher muscle weight compared to the controls at day 14 (Fig 4M), which further confirmed that they underwent a more efficient regeneration. Moreover, the AMPK pathway responses downstream of mGPDH were also activated by AAV‐mGPDH (Fig 4N), corroborating our *in vitro* mechanistic findings. In addition, previous studies have shown that inflammatory signaling is an important player in the regulation of tissue repair and regeneration (De Bleecker & Engel, 1994). However, although mGPDH ablation (mGPDH^−/−^ mice) or overexpression (AAV‐mGPDH) showed relatively decreased or increased trends of cytokine genes, respectively, their expressions were not statistically significant with the exception of IL‐1β in the mGPDH^−/−^ mice (Fig EV5A–C).

## Discussion

Both obese and diabetic patients have been proven to suffer from delayed skeletal muscle regeneration, which led to impaired muscle function and a poor prognosis (Hu *et al*, 2010; Fu *et al*, 2016). The present study identified mGPDH as a pivotal regulator of myoblast differentiation that contributes to the process of skeletal muscle regeneration. The mechanical studies indicated that the effects were mainly through CaMKKβ/AMPK‐controlled mitochondrial biogenesis. Importantly, the mGPDH expression of skeletal muscle was reduced in patients and animal models of obesity and diabetes, and rescuing mGPDH expression led to a significant improvement in muscle regeneration.

Our findings suggest that mGPDH is a critical regulator of skeletal muscle regeneration through the regulation of CaMKKβ/AMPK‐controlled mitochondrial biogenesis. mGPDH deletion attenuated skeletal muscle regeneration *in vitro* and *in vivo*, while its overexpression ameliorated dystrophic pathology in mdx mice. Interestingly, our study showed an increased basal expression of myogenin in mdx mouse muscles compared with normal mice, which is consistent with previous observations (Turk *et al*, 2005). This result indicates an activated regeneration process; however, it remains insufficient to compensate due to persistent damage induced by the *Dmd* gene mutation in mdx mice (Barton *et al*, 2002; Duddy *et al*, 2015; Novak *et al*, 2017). Therefore, as shown in our study, the basal level of mGPDH is also increased in mdx mice. The regulatory role of mitochondrial biogenesis on muscle regeneration has been proven by several studies (Cerletti *et al*, 2012; Stein & Imai, 2014; Zhang *et al*, 2014; Katajisto *et al*, 2015). AMPK, as the most important regulator of mitochondrial function, modulates muscle regeneration *via* mitochondrial biogenesis as previously reported (Woldt *et al*, 2013). Our results demonstrated that mGPDH regulates skeletal muscle regeneration through AMPK‐controlled mitochondrial biogenesis, which may be a new upstream regulation mechanism of AMPK on muscle regeneration. Our study also suggests that the effect of mGPDH is mainly through CaMKKβ‐triggered AMPK activation, while LKB1 does not seem to participate in this process. As a calmodulin‐dependent kinase, CaMKKβ activation depends on the intracellular Ca^2+^ concentration (Marcelo *et al*, 2016). Previous studies have reported that mGPDH regulates the intracellular Ca^2+^ concentration in pancreatic beta cells (Eto *et al*, 1999a,b), which further confirmed our observation regarding the regulation of mGPDH on CaMKKβ. Notably, although CaMKKβ and LKB1 are known to be two major AMPKKs (Hawley *et al*, 2005; Kahn *et al*, 2005; Woods *et al*, 2005), the participation of other AMPKKs, such as AMP, PI3K, and PKC, cannot be completely ruled out (Zou *et al*, 2003; Nishino *et al*, 2004; Oakhill *et al*, 2011). Our study strengthened previous observations regarding the impact of mitochondrial biogenesis on myoblast differentiation and muscle regeneration. An enhanced mitochondrial mass and/or function promoted myoblast differentiation and muscle regeneration, while attenuated mitochondrial biogenesis inhibited them by manipulating the myogenic regulatory factors. Our results are consistent with these findings and further confirmed an important role of mitochondrial biogenesis on functional muscle regeneration (Cerletti *et al*, 2012; Duguez *et al*, 2012; Varaljai *et al*, 2015).

Our study provides a potential therapeutic target for skeletal muscle regeneration impairment during obesity and diabetes. Deficient mGPDH expression was identified in the skeletal muscles of patients and animal models of obesity and diabetes. Rescuing mGPDH expression substantially promotes muscle regeneration in HFD, *ob*/*ob*, and STZ mice, which indicates the therapeutic potential of targeting mGPDH. Previous studies have suggested that several common treatments for obesity and diabetes, such as moderate exercise, metformin, and GLP‐1 agonists, are capable of promoting muscle regeneration by controlling mitochondrial biogenesis (Tong *et al*, 2011; Wu *et al*, 2011; Yamamoto *et al*, 2013). Therefore, further investigations are required to explore the potential relationships between mGPDH and the effects of these therapies on muscle regeneration. Moreover, the application of mGPDH activators for these diseases should be investigated in the future.

Collectively, our study revealed a novel functional role of mGPDH in regulating myogenic differentiation and skeletal muscle regeneration. In addition, activation of the mGPDH/AMPK/mitochondrial biogenesis pathway in skeletal muscle might be a mechanism of importance for ameliorating muscular frailty during obesity and diabetes based on our *in vitro* explorations and animal model recapitulations. Direct therapeutic targeting of mGPDH may also have therapeutic potential.

## Materials and Methods

### Cell culture

C2C12 myocytes were purchased from the Cell Bank of the Chinese Academy of Sciences and were maintained at subconfluent densities in growth medium consisting of Dulbecco's modified Eagle's medium (DMEM; Gibco) supplemented with 10% fetal bovine serum (FBS; Gibco) in a 5% CO_2_ incubator at 37°C. To induce myogenic differentiation, cells were grown to 95% confluence in growth medium and then cultured in differentiation medium composed of DMEM and 2% horse serum (Gibco). The differentiation medium was changed every 48 h. All cell identities were confirmed and cultured as recommended by the supplier. Mycoplasma determination was performed by Shanghai Biowing Applied Biotechnology Co., and no mycoplasma contamination was identified in these cells.

### Cell transfection and treatment

For the knockdown, the cells were seeded in 6‐well culture plates and transiently transfected with 50 ng per well of siRNA oligonucleotides with RNAiMAX (Life Technology) according to the manufacturer's instructions. mGPDH‐specific, LKB1‐specific, and corresponding negative‐control siRNAs were synthesized by Qiagen, and the sequences are presented in Appendix Table S2. For overexpression, cells were seeded in 6‐well culture plates and transfected with 1 μg of plasmid using Lipofectamine 3000 reagent (Invitrogen) according to the manufacturer's instructions. Plasmids of the pRP‐mGPDH and control vector were generated by VectorBuilder. Compound C and STO‐609 were purchased from Sigma‐Aldrich. BAPTA‐AM was purchased from TCI.

### Glucose uptake

Cells were serum‐starved for 3 h in culture medium (no glucose) and subsequently incubated with 100 μM of fluorescent deoxyglucose 2‐NBDG (Invitrogen) with or without 100 nM of insulin (Biosharp) for 1 h. The cells were washed and harvested in ice‐cold PBS. The fluorescence intensity was determined using a flow cytometer (MoFlo XDP; Beckman Coulter) at excitation and emission wavelengths of 485 and 538 nm, respectively.

### Cell mitochondrial protein isolation

The mitochondria of cells were isolated using a Mitochondria Isolation Kit (Beyotime) according to the manufacturer's recommendation. Briefly, the cells were collected, re‐suspended, and homogenized in 1 ml of mitochondrial separation reagent supplemented with PMSF, and the suspension was then centrifuged at 600 *g* for 10 min at 4°C to remove nuclei and cell debris. The supernatant was re‐centrifuged at 11,000 *g* for 10 min at 4°C, and the remaining pellet contained the mitochondria. Mitochondrial lysis solution (50 μl) was added to break down the mitochondrial proteins, and protein concentrations were detected with the BCA Protein Assay Kit (Beyotime).

### Cell cycle and apoptosis analyses

For the cell cycle analysis, the cells were detached by trypsin and fixed in 70% ethanol overnight. The cells were washed and re‐suspended in 0.5 ml of PBS that contained 100 μg/ml of RNase A and 5 μg/ml of propidium iodide (PI) for 30 min. The DNA contents were measured by flow cytometry. Apoptosis was detected using the FITC Annexin V Apoptosis Detection Kit (Becton Dickinson) according to the manufacturer's instructions. Briefly, cells were harvested and washed twice with ice‐cold PBS, re‐suspended in 100 μl of binding buffer, and incubated with 5 μl of Annexin V–FITC and 5 μl of PI. After a 30‐min incubation at room temperature in the dark, 400 μl of binding buffer was added to each tube, and the samples were immediately analyzed via flow cytometry.

### Cell oxygen consumption rate (OCR) measurement

The cellular oxidation state was measured using a Seahorse XF96 extracellular flux analyzer (Seahorse Biosciences) according to the manufacturer's protocol. In brief, 10,000 cells were seeded in each well of XF 96‐well microplates (Seahorse Bioscience). The final concentrations of the mitochondrial inhibitors were 1 μM of oligomycin, 3 μM of FCCP, and 0.5 of μM rotenone. Basal respiration indicates the baseline oxygen consumption reading prior to compound injection. Maximal respiration represents the maximum OCR measurement value after FCCP injection. After detection, the cell protein concentrations were assessed and the OCR was adjusted accordingly.

### Mitochondrial content

Total DNA was extracted using a DNA purification kit (Promega) according to the manufacturer's instructions. The mitochondrial DNA copy number was determined by real‐time PCR using primers specific for the mitochondrial cytochrome c oxidase subunit 2 (COX2) gene as previously described (Price *et al*, 2012) and was normalized to the nuclear copy number using primers specific for the ribosomal protein s18 (S18) nuclear gene. A complete list of the primer sequences is presented in Appendix Table S2.

### NAD^+^/NADH measurements and PGC1α acetylation assay

The NAD^+^ and NADH contents were measured using a NAD^+^/NADH Assay Kit (Beyotime) according to the manufacturer's recommendation. The amounts of PGC1α acetylation were performed as previously described (Woldt *et al*, 2013). Briefly, cells were transfected with plasmid of Flag‐PGC1α expression vector, and where indicated, mGPDH siRNA or plasmid was used to knock down or overexpress mGPDH as indicated. The cells were harvested, and the PGC1α acetylation was determined by immunoprecipitation of lysates with anti‐PGC1α antibody (Novus NBP1‐04676, 2 μg per sample), followed by Western blot analysis using antibodies to acetylated lysine (1:500, Santa Cruz, sc‐32268). The input was blotted with antibody to Flag‐epitope tag (1:1,000, Cell Signaling Technology, 8146s).

### Mice

All mouse experiments were performed in accordance with protocols approved by the Laboratory Animal Welfare and Ethics Committee of the Third Military Medical University. mGPDH^−/−^, *ob*/*ob*, and mdx mice were purchased from the Model Animal Research Center of Nanjing University, *db*/*db* mice were a gift from Professor Gangyi Yang (Chongqing Medical University), and C57BL/6J mice were purchased from Beijing HFK Bioscience Co. Male mice were used in all experiments with the exception of mGPDH^−/−^ and their littermate controls, where male and female mice were equally used. All mice were housed with a 12‐h dark/light cycle with food and water *ad libitum* and were randomly allocated to the indicated groups. Blinding was used for the analysis of all animal experiments with the exception of the qRT–PCR and immunoblot analyses.

### HFD and STZ mouse models

C57BL/6J mice were placed on a high‐fat diet [HFD, 60 kcal% from fat, purchased from Research Diets (New Brunswick)] at the age of 5 weeks and were maintained on the same diet for 12 weeks. Diabetes was induced via the intraperitoneal (i.p.) injection of streptozotocin (STZ, 50 mg/kg, pH 4.5, dissolved in sodium citrate; Sigma) for five consecutive days. Two weeks after STZ injection, the fasting plasma glucose (FPG, 8 h fast) was measured, and the mice with an FPG level greater than 250 mg/dl (13.9 mmol/l) were considered diabetic (Zheng *et al*, 2011).

### Skeletal muscle injury

Animals were anesthetized using pentobarbital sodium (50 mg/kg) *via* i.p. injection. To induce muscle injury, 50 μl of 10 μM cardiotoxin (CTX; Sigma) was injected using an insulin syringe (U‐100; Becton Dickinson) into muscles (Rozo *et al*, 2016).

### AAV gene transfer

To avoid an *in vivo* immune response, adeno‐associated virus (AAV)‐9, which is specific to skeletal muscle with broad myofiber‐specific tropism, was used (Kotterman & Schaffer, 2014; Riaz *et al*, 2015). The recombinant mGPDH‐encoding AAV9 and the control vectors (AAV9‐GFP) were generated by GENE Company. After anesthetization, AAV particles (8 × 10^10^ v.g.) were singly injected into the gastrocnemius (GA) muscles. For tail vein injection, the volume of AAV9 was 1.2 × 10^11^ v.g.

### Treadmill test

The treadmill test was conducted using the ZH‐PT animal running experiment (Zhenghua Biologic Apparatus Facilities) at 15 degrees downhill. Mice were warmed up at 5 m/min for 2 min, and then ran on the treadmill at 7 m/min for 2 min, 8 m/min for 2 min, and 12 m/min for 5 min. The speed was subsequently increased by 1 m/min to a final speed of 20 m/min. Exhaustion was defined as the inability of the animal to remain on the treadmill despite electrical prodding for 10 s. The running time and distance were calculated.

### H&E, Masson's trichrome, and immunohistochemical analyses

Hematoxylin and eosin (H&E), Masson's trichrome, and immunohistochemical (IHC) staining were performed as previously described (Zheng *et al*, 2011). Regenerating myofibers were defined as myofibers that contained central nuclei, and the cross‐sectional area (CSA) of the regenerating myofibers was determined using ImageJ (NIH) software. Nuclei per fiber were counted as the average number of central nuclei in each regenerating myofiber.

### Western blot and antibodies

For protein extraction, the cells were lysed in sample buffer [50 mM of Tris–HCl (pH 6.8), 2% SDS, 10% glycerol, 100 mM of dithiothreitol, and 0.1% bromophenol blue], tissue lysates were prepared as previously described (Wang *et al*, 2016), and protein concentrations were measured using the BCA Protein Assay Kit (Beyotime). Extracted protein lysates were resolved by SDS–PAGE and immunoblotted with the indicated primary antibodies and their corresponding HRP‐conjugated secondary antibodies. Blots were developed with chemiluminescent HRP substrate (Millipore) and imaged using a Fusion FX5s system (Vilber Lourmat). The following antibodies were used: mGPDH (1:1,000, sc‐390830), Myogenin (1:1,000, sc‐12732), cGPDH (1:500, sc‐376219), IGF‐1R (1:1,000, sc‐81464), GAPDH (1:10,000, sc‐20357), β‐actin (1:2,000, sc‐47778), and c‐myc (1:1,000, sc‐42) from Santa Cruz Biotechnology; MyHC (1:2,000, M4276) from Sigma; Akt (1:1,000, #4691), p‐Akt (Thr308, 1:1,000, #13038), IRS1 (1:1,000, #2382), p‐IRS1 (Ser307, 1:1,000, #2381), VDAC (1:1,000, #4866), Cyt c (1:1,000, #4280), AMPKα (1:2,000, #2532), p‐AMPKα (Thr172, 1:2,000, #2535), ACC (1:1,000, #3676), p‐ACC (Ser79, 1:1,000, #11818), Flag (1:1,000, #8146), and LC3B (1:1,000, #2775) from Cell Signaling Technology; PGC1α (1:1,000, ab54481) and mGPDH (1:10,000, ab188585) from Abcam; and COX IV (1:500, AC610) from Beyotime. The relative band intensities were quantified using the Fusion FX5s system (Vilber Lourmat).

### Quantitative real‐time PCR (qRT–PCR)

Total RNA was isolated from cells or tissues using RNAiso Plus (Takara), according to the manufacturer's instructions. The RNA quality was assessed on a NanoDrop 2000 (Thermo), where the 260/280 ratio was obtained. Samples with a ratio of 1.8–2.0 were processed for downstream gene analysis. 1,000 ng of RNA was reverse‐transcribed into cDNA using a PrimeScript RT Reagent Kit with gDNA Eraser (Takara) according to the manufacturer's protocol. qRT–PCR was performed with SYBR Premix Ex Taq II (Takara) on a Bio‐Applied Biosystems 7300 (Life Technology). The results were analyzed using the comparative cycle threshold (CT) method. The primers are presented in Appendix Table S2.

### mGPDH activity assay

The mGPDH activity was measured using mitochondria, which were separated from total homogenate according to the manufacturer's recommendation of the Mitochondria Isolation Kit (Beyotime). The activity was detected using 2,6‐dichloroindophenol (DCIP; Sigma) as the electron acceptor and measuring the loss of absorbance at 600 nm (reaction buffer consisted of 50 mM of KH_2_PO_4_/K_2_HPO_4_ buffer (pH 7.5), 9.3 μM of antimycin A, 5 μM of rotenone, and 50 μM of DCIP; reaction conducted at 37°C).

### Immunofluorescence staining

Cells and tissue sections were fixed in 4% paraformaldehyde for 30 min, permeabilized with 0.1–0.2% Triton X‐100 (Sigma) at room temperature (RT) for 10–15 min, blocked with 5% BSA for 30 min at RT, and incubated with the indicated primary antibodies (1:50–1:100) overnight at 4°C, followed by their corresponding secondary antibodies for 1 h at RT. Nuclei were stained with DAPI (Beyotime) for 5 min, and the coverslips were mounted with Fluoromount (BOSTER); images were acquired using a fluorescence microscope (Olympus cellSens standard 1.15). The fusion index was calculated as the ratio of the number of nuclei incorporated into myotubes (> 2 nuclei) to the total number of nuclei. Nuclei were counted from five images/dish using ImageJ. Nuclear number assays were determined by calculating the percentage of nuclei incorporated into the MyHC‐positive myotubes with the indicated number of nuclei.

### Human clinical studies

All experimental protocols were approved by the Ethics Committee of Xinqiao Hospital, Third Military Medical University, and were registered online (Clinical trial register no. ChiCTR‐ROC‐17010719), and conformed to the principles set out in the WMA Declaration of Helsinki and the Department of Health and Human Services Belmont Report. All participants provided written informed consent. A total of 11 obese patients and 18 normal subjects participated in this study. Obesity was defined according to the WHO Western Pacific Regional Office definition (Wen *et al*, 2009). Biopsies of GA muscles were obtained from all participants, and the clinical characteristics are presented in Appendix Table S1.

### Statistical analyses

All data were analyzed using GraphPad Prism 7 (Macintosh). Quantitative values are presented as the mean ± s.e.m. Statistical differences between two experimental groups were analyzed using two‐tailed Student's *t*‐test. The mean IHC scores were analyzed using the Wilcoxon test. The distribution of nuclei per myotube and the fiber cross‐sectional area was analyzed using the Kolmogorov–Smirnov test. *P*‐values < 0.05 were considered significant. Sample sizes were estimated on the basis of our previous experiences (Zheng *et al*, 2011; Wang *et al*, 2016), and no samples were excluded from the study. All *P*‐values for the main figures, expanded view figures, and appendix figures are presented in Dataset EV1.

## Author contributions

HQ, XL, YZ, QL, and LZ: acquisition of data, analysis and interpretation of data, and statistical analysis; HQ, YZ, and QL: drafting of the manuscript; HQ, XL, XX, YW, RZ, QT, HW, and JX: analysis and interpretation of data; ZZ, GY, ZL, and HD: critical revision of the manuscript for important intellectual content; HZ: study concept and design, analysis and interpretation of data, drafting of the manuscript, critical revision of the manuscript for important intellectual content, obtaining study funding, and study supervision.

## Conflict of interest

The authors declare that they have no conflict of interest.

**The paper explained**

**Problem**

While adult mammalian skeletal muscle is stable due to its post‐mitotic nature, muscle regeneration serves a critical role in maintaining functional fitness throughout life. During certain diseases, such as the modern pandemics of obesity and diabetes, the regeneration process becomes impaired, which leads to the loss of muscle function and contributes to the global burden of these diseases. However, the underlying mechanisms remain poorly defined.

**Results**

In this study, we report that mitochondrial glycerol 3‐phosphate dehydrogenase (mGPDH, also referred to as *GPD2*) is a critical regulator for skeletal muscle regeneration. Specifically, mGPDH promotes myogenic gene expression and myoblast differentiation by controlling mitochondrial biogenesis via CaMKKβ/AMPK. mGPDH deletion attenuated skeletal muscle regeneration *in vitro* and *in vivo*, while activating mGPDH ameliorated dystrophic pathology in mdx mice. Moreover, skeletal muscle mGPDH expression was reduced in patients and animal models of obesity and diabetes, and its restoration resulted in improved muscle regeneration.

**Impact**

Our study indicates a novel characteristic of mGPDH in regulating myogenic differentiation and identifies a potential complementary strategy for enhancing muscle regeneration and ameliorating muscle pathology. In addition, activation of the mGPDH/AMPK/mitochondrial biogenesis pathway of skeletal muscle may represent a new mechanism for treatment during obesity and diabetes.

## Supporting information



Appendix

Expanded View Figures PDF

Review Process File

Dataset EV1

Source Data for Figure 1

Source Data for Figure 2

Source Data for Figure 3

Source Data for Figure 4

## References

[emmm201809390-bib-0001] Aragno M , Mastrocola R , Catalano MG , Brignardello E , Danni O , Boccuzzi G (2004) Oxidative stress impairs skeletal muscle repair in diabetic rats. Diabetes 53: 1082–1088 15047625 10.2337/diabetes.53.4.1082

[emmm201809390-bib-0002] Barton ER , Morris L , Musaro A , Rosenthal N , Sweeney HL (2002) Muscle‐specific expression of insulin‐like growth factor I counters muscle decline in mdx mice. J Cell Biol 157: 137–148 11927606 10.1083/jcb.200108071 PMC2173262

[emmm201809390-bib-0003] Baur JA , Birnbaum MJ (2014) Control of gluconeogenesis by metformin: does redox trump energy charge? Cell Metab 20: 197–199 25100057 10.1016/j.cmet.2014.07.013 PMC4154964

[emmm201809390-bib-0004] Benoit B , Meugnier E , Castelli M , Chanon S , Vieille‐Marchiset A , Durand C , Bendridi N , Pesenti S , Monternier PA , Durieux AC *et al* (2017) Fibroblast growth factor 19 regulates skeletal muscle mass and ameliorates muscle wasting in mice. Nat Med 23: 990–996 28650457 10.1038/nm.4363

[emmm201809390-bib-0005] Bi P , Ramirez‐Martinez A , Li H , Cannavino J , McAnally JR , Shelton JM , Sanchez‐Ortiz E , Bassel‐Duby R , Olson EN (2017) Control of muscle formation by the fusogenic micropeptide myomixer. Science 356: 323–327 28386024 10.1126/science.aam9361 PMC5502127

[emmm201809390-bib-0006] Cerletti M , Jang YC , Finley LW , Haigis MC , Wagers AJ (2012) Short‐term calorie restriction enhances skeletal muscle stem cell function. Cell Stem Cell 10: 515–519 22560075 10.1016/j.stem.2012.04.002 PMC3561899

[emmm201809390-bib-0007] Charge SB , Rudnicki MA (2004) Cellular and molecular regulation of muscle regeneration. Physiol Rev 84: 209–238 14715915 10.1152/physrev.00019.2003

[emmm201809390-bib-0008] Chiu CY , Yang RS , Sheu ML , Chan DC , Yang TH , Tsai KS , Chiang CK , Liu SH (2016) Advanced glycation end‐products induce skeletal muscle atrophy and dysfunction in diabetic mice via a RAGE‐mediated, AMPK‐down‐regulated, Akt pathway. J Pathol 238: 470–482 26586640 10.1002/path.4674

[emmm201809390-bib-0009] De Bleecker JL , Engel AG (1994) Expression of cell adhesion molecules in inflammatory myopathies and Duchenne dystrophy. J Neuropathol Exp Neurol 53: 369–376 8021710 10.1097/00005072-199407000-00008

[emmm201809390-bib-0010] Duddy W , Duguez S , Johnston H , Cohen TV , Phadke A , Gordish‐Dressman H , Nagaraju K , Gnocchi V , Low S , Partridge T (2015) Muscular dystrophy in the mdx mouse is a severe myopathy compounded by hypotrophy, hypertrophy and hyperplasia. Skelet Muscle 5: 16 25987977 10.1186/s13395-015-0041-y PMC4434871

[emmm201809390-bib-0011] Duguez S , Duddy WJ , Gnocchi V , Bowe J , Dadgar S , Partridge TA (2012) Atmospheric oxygen tension slows myoblast proliferation via mitochondrial activation. PLoS One 7: e43853 22937109 10.1371/journal.pone.0043853 PMC3427224

[emmm201809390-bib-0012] Durko M , Allen C , Nalbantoglu J , Karpati G (2010) CT‐GalNAc transferase overexpression in adult mice is associated with extrasynaptic utrophin in skeletal muscle fibres. J Muscle Res Cell Motil 31: 181–193 20706865 10.1007/s10974-010-9222-9

[emmm201809390-bib-0013] Eto K , Suga S , Wakui M , Tsubamoto Y , Terauchi Y , Taka J , Aizawa S , Noda M , Kimura S , Kasai H *et al* (1999a) NADH shuttle system regulates K(ATP) channel‐dependent pathway and steps distal to cytosolic Ca(2+) concentration elevation in glucose‐induced insulin secretion. J Biol Chem 274: 25386–25392 10464266 10.1074/jbc.274.36.25386

[emmm201809390-bib-0014] Eto K , Tsubamoto Y , Terauchi Y , Sugiyama T , Kishimoto T , Takahashi N , Yamauchi N , Kubota N , Murayama S , Aizawa T *et al* (1999b) Role of NADH shuttle system in glucose‐induced activation of mitochondrial metabolism and insulin secretion. Science 283: 981–985 9974390 10.1126/science.283.5404.981

[emmm201809390-bib-0015] Fieni F , Johnson DE , Hudmon A , Kirichok Y (2014) Mitochondrial Ca^2+^ uniporter and CaMKII in heart. Nature 513: 1–2 10.1038/nature13626 PMC4476531 25254480

[emmm201809390-bib-0016] Fu X , Zhu M , Zhang S , Foretz M , Viollet B , Du M (2016) Obesity impairs skeletal muscle regeneration through inhibition of AMPK. Diabetes 65: 188–200 26384382 10.2337/db15-0647 PMC4686944

[emmm201809390-bib-0017] Ganitkevich VY (2003) The role of mitochondria in cytoplasmic Ca^2+^ cycling. Exp Physiol 88: 91–97 12525858 10.1113/eph8802504

[emmm201809390-bib-0018] Garcia‐Prat L , Martinez‐Vicente M , Perdiguero E , Ortet L , Rodriguez‐Ubreva J , Rebollo E , Ruiz‐Bonilla V , Gutarra S , Ballestar E , Serrano AL *et al* (2016) Autophagy maintains stemness by preventing senescence. Nature 529: 37–42 26738589 10.1038/nature16187

[emmm201809390-bib-0019] Gomes AP , Price NL , Ling AJY , Moslehi JJ , Montgomery MK , Rajman L , White JP , Teodoro JS , Wrann CD , Hubbard BP (2013) Declining NAD^+^ induces a pseudohypoxic state disrupting nuclear‐mitochondrial communication during aging. Cell 155: 1624–1638 24360282 10.1016/j.cell.2013.11.037 PMC4076149

[emmm201809390-bib-0020] Hawley SA , Pan DA , Mustard KJ , Ross L , Bain J , Edelman AM , Frenguelli BG , Hardie DG (2005) Calmodulin‐dependent protein kinase kinase‐beta is an alternative upstream kinase for AMP‐activated protein kinase. Cell Metab 2: 9–19 16054095 10.1016/j.cmet.2005.05.009

[emmm201809390-bib-0021] Hochreiter‐Hufford AE , Lee CS , Kinchen JM , Sokolowski JD , Arandjelovic S , Call JA , Klibanov AL , Yan Z , Mandell JW , Ravichandran KS (2013) Phosphatidylserine receptor BAI1 and apoptotic cells as new promoters of myoblast fusion. Nature 497: 263–267 23615608 10.1038/nature12135 PMC3773542

[emmm201809390-bib-0022] Hu Z , Wang H , Lee IH , Modi S , Wang X , Du J , Mitch WE (2010) PTEN inhibition improves muscle regeneration in mice fed a high‐fat diet. Diabetes 59: 1312–1320 20200318 10.2337/db09-1155 PMC2874691

[emmm201809390-bib-0023] Iwabu M , Yamauchi T , Okada‐Iwabu M , Sato K , Nakagawa T , Funata M , Yamaguchi M , Namiki S , Nakayama R , Tabata M *et al* (2010) Adiponectin and AdipoR1 regulate PGC‐1alpha and mitochondria by Ca(2+) and AMPK/SIRT1. Nature 464: 1313–1319 20357764 10.1038/nature08991

[emmm201809390-bib-0024] Kahn BB , Alquier T , Carling D , Hardie DG (2005) AMP‐activated protein kinase: ancient energy gauge provides clues to modern understanding of metabolism. Cell Metab 1: 15–25 16054041 10.1016/j.cmet.2004.12.003

[emmm201809390-bib-0025] Katajisto P , Dohla J , Chaffer CL , Pentinmikko N , Marjanovic N , Iqbal S , Zoncu R , Chen W , Weinberg RA , Sabatini DM (2015) Stem cells. Asymmetric apportioning of aged mitochondria between daughter cells is required for stemness. Science 348: 340–343 25837514 10.1126/science.1260384 PMC4405120

[emmm201809390-bib-0026] Kim YK , Ha HH , Lee JS , Bi X , Ahn YH , Hajar S , Lee JJ , Chang YT (2010) Control of muscle differentiation by a mitochondria‐targeted fluorophore. J Am Chem Soc 132: 576–579 20020712 10.1021/ja906862g

[emmm201809390-bib-0027] Kotterman MA , Schaffer DV (2014) Engineering adeno‐associated viruses for clinical gene therapy. Nat Rev Genet 15: 445–451 24840552 10.1038/nrg3742 PMC4393649

[emmm201809390-bib-0028] Lee DC , Shook RP , Drenowatz C , Blair SN (2016) Physical activity and sarcopenic obesity: definition, assessment, prevalence and mechanism. Future Sci OA 2: FSO127 28031974 10.4155/fsoa-2016-0028 PMC5137918

[emmm201809390-bib-0029] Lin YY , Kiihl S , Suhail Y , Liu SY , Chou YH , Kuang Z , Lu JY , Khor CN , Lin CL , Bader JS *et al* (2012) Functional dissection of lysine deacetylases reveals that HDAC1 and p300 regulate AMPK. Nature 482: 251–255 22318606 10.1038/nature10804 PMC3277212

[emmm201809390-bib-0030] Liu N , Williams AH , Maxeiner JM , Bezprozvannaya S , Shelton JM , Richardson JA , Bassel‐Duby R , Olson EN (2012) microRNA‐206 promotes skeletal muscle regeneration and delays progression of Duchenne muscular dystrophy in mice. J Clin Invest 122: 2054–2065 22546853 10.1172/JCI62656 PMC3366415

[emmm201809390-bib-0031] Madiraju AK , Erion DM , Rahimi Y , Zhang XM , Braddock DT , Albright RA , Prigaro BJ , Wood JL , Bhanot S , MacDonald MJ *et al* (2014) Metformin suppresses gluconeogenesis by inhibiting mitochondrial glycerophosphate dehydrogenase. Nature 510: 542–546 24847880 10.1038/nature13270 PMC4074244

[emmm201809390-bib-0032] Marcelo KL , Means AR , York B (2016) The Ca(2+)/calmodulin/CaMKK2 axis: nature's metabolic CaMshaft. Trends Endocrinol Metab 27: 706 27449752 10.1016/j.tem.2016.06.001 PMC5035586

[emmm201809390-bib-0033] Meng ZX , Li S , Wang L , Ko HJ , Lee Y , Jung DY , Okutsu M , Yan Z , Kim JK , Lin JD (2013) Baf60c drives glycolytic metabolism in the muscle and improves systemic glucose homeostasis through Deptor‐mediated Akt activation. Nat Med 19: 640–645 23563706 10.1038/nm.3144 PMC3650110

[emmm201809390-bib-0034] Miner JH , Wold BJ (1991) c‐myc inhibition of MyoD and myogenin‐initiated myogenic differentiation. Mol Cell Biol 11: 2842–2851 1850105 10.1128/mcb.11.5.2842-2851.1991 PMC360069

[emmm201809390-bib-0035] Mottillo EP , Desjardins EM , Crane JD , Smith BK , Green AE , Ducommun S , Henriksen TI , Rebalka IA , Razi A , Sakamoto K *et al* (2016) Lack of adipocyte AMPK exacerbates insulin resistance and hepatic steatosis through brown and beige adipose tissue function. Cell Metab 24: 118–129 27411013 10.1016/j.cmet.2016.06.006 PMC5239668

[emmm201809390-bib-0036] Mounier R , Theret M , Arnold L , Cuvellier S , Bultot L , Goransson O , Sanz N , Ferry A , Sakamoto K , Foretz M *et al* (2013) AMPKalpha1 regulates macrophage skewing at the time of resolution of inflammation during skeletal muscle regeneration. Cell Metab 18: 251–264 23931756 10.1016/j.cmet.2013.06.017

[emmm201809390-bib-0037] Mracek T , Drahota Z , Houstek J (2013) The function and the role of the mitochondrial glycerol‐3‐phosphate dehydrogenase in mammalian tissues. Biochem Biophys Acta 1827: 401–410 23220394 10.1016/j.bbabio.2012.11.014

[emmm201809390-bib-0038] Musaro A , McCullagh K , Paul A , Houghton L , Dobrowolny G , Molinaro M , Barton ER , Sweeney HL , Rosenthal N (2001) Localized Igf‐1 transgene expression sustains hypertrophy and regeneration in senescent skeletal muscle. Nat Genet 27: 195–200 11175789 10.1038/84839

[emmm201809390-bib-0039] Nishino Y , Miura T , Miki T , Sakamoto J , Nakamura Y , Ikeda Y , Kobayashi H , Shimamoto K (2004) Ischemic preconditioning activates AMPK in a PKC‐dependent manner and induces GLUT4 up‐regulation in the late phase of cardioprotection. Cardiovasc Res 61: 610–619 14962491 10.1016/j.cardiores.2003.10.022

[emmm201809390-bib-0040] Novak JS , Hogarth MW , Boehler JF , Nearing M , Vila MC , Heredia R , Fiorillo AA , Zhang A , Hathout Y , Hoffman EP *et al* (2017) Myoblasts and macrophages are required for therapeutic morpholino antisense oligonucleotide delivery to dystrophic muscle. Nat Commun 8: 941 29038471 10.1038/s41467-017-00924-7 PMC5643396

[emmm201809390-bib-0041] Oakhill JS , Steel R , Chen ZP , Scott JW , Ling N , Tam S , Kemp BE (2011) AMPK is a direct adenylate charge‐regulated protein kinase. Science 332: 1433–1435 21680840 10.1126/science.1200094

[emmm201809390-bib-0042] Park SY , Yun Y , Lim JS , Kim MJ , Kim SY , Kim JE , Kim IS (2016) Stabilin‐2 modulates the efficiency of myoblast fusion during myogenic differentiation and muscle regeneration. Nat Commun 7: 10871 26972991 10.1038/ncomms10871 PMC4793076

[emmm201809390-bib-0043] Price NL , Gomes AP , Ling AJ , Duarte FV , Martin‐Montalvo A , North BJ , Agarwal B , Ye L , Ramadori G , Teodoro JS *et al* (2012) SIRT1 is required for AMPK activation and the beneficial effects of resveratrol on mitochondrial function. Cell Metab 15: 675–690 22560220 10.1016/j.cmet.2012.04.003 PMC3545644

[emmm201809390-bib-0044] Ravel‐Chapuis A , Crawford TE , Blais‐Crépeau ML , Bélanger G , Richer CT , Jasmin BJ (2014) The RNA‐binding protein Staufen1 impairs myogenic differentiation via a c‐myc‐dependent mechanism. Mol Biol Cell 25: 3765 25208565 10.1091/mbc.E14-04-0895 PMC4230783

[emmm201809390-bib-0045] Riaz M , Raz Y , Moloney EB , van Putten M , Krom YD , van der Maarel SM , Verhaagen J , Raz V (2015) Differential myofiber‐type transduction preference of adeno‐associated virus serotypes 6 and 9. Skelet Muscle 5: 37 26561520 10.1186/s13395-015-0064-4 PMC4641337

[emmm201809390-bib-0046] Rozo M , Li L , Fan CM (2016) Targeting beta1‐integrin signaling enhances regeneration in aged and dystrophic muscle in mice. Nat Med 22: 889–896 27376575 10.1038/nm.4116 PMC4974124

[emmm201809390-bib-0047] Seyer P , Grandemange S , Busson M , Carazo A , Gamaléri F , Pessemesse L , Casas F , Cabello G , Wrutniak‐Cabello C (2010) Mitochondrial activity regulates myoblast differentiation by control of c‐Myc expression. J Cell Physiol 207: 75–86 10.1002/jcp.20539 16261590

[emmm201809390-bib-0048] Stefani DD , Rizzuto R , Pozzan T (2016) Enjoy the trip: calcium in mitochondria back and forth. Annu Rev Biochem 85: 161–192 27145841 10.1146/annurev-biochem-060614-034216

[emmm201809390-bib-0049] Stein LR , Imai S (2014) Specific ablation of Nampt in adult neural stem cells recapitulates their functional defects during aging. EMBO J 33: 1321–1340 24811750 10.1002/embj.201386917 PMC4194122

[emmm201809390-bib-0050] Tong JF , Yan X , Zhao JX , Zhu MJ , Nathanielsz PW , Du M (2011) Metformin mitigates the impaired development of skeletal muscle in the offspring of obese mice. Nutr Diabetes 1: e7 23449382 10.1038/nutd.2011.3 PMC3302133

[emmm201809390-bib-0051] Turk R , Sterrenburg E , de Meijer EJ , van Ommen GJ , den Dunnen JT , ‘t Hoen PA (2005) Muscle regeneration in dystrophin‐deficient mdx mice studied by gene expression profiling. BMC Genom 6: 98 10.1186/1471-2164-6-98 PMC1190170 16011810

[emmm201809390-bib-0052] Varaljai R , Islam AB , Beshiri ML , Rehman J , Lopez‐Bigas N , Benevolenskaya EV (2015) Increased mitochondrial function downstream from KDM5A histone demethylase rescues differentiation in pRB‐deficient cells. Genes Dev 29: 1817–1834 26314709 10.1101/gad.264036.115 PMC4573855

[emmm201809390-bib-0053] Wang H , Liu X , Long M , Huang Y , Zhang L , Zhang R , Zheng Y , Liao X , Wang Y , Liao Q *et al* (2016) NRF2 activation by antioxidant antidiabetic agents accelerates tumor metastasis. Sci Transl Med 8: 334ra351 10.1126/scitranslmed.aad6095 27075625

[emmm201809390-bib-0054] Wen CP , David Cheng TY , Tsai SP , Chan HT , Hsu HL , Hsu CC , Eriksen MP (2009) Are Asians at greater mortality risks for being overweight than Caucasians? Redefining obesity for Asians. Public Health Nutr 12: 497–506 18547457 10.1017/S1368980008002802

[emmm201809390-bib-0055] Woldt E , Sebti Y , Solt LA , Duhem C , Lancel S , Eeckhoute J , Hesselink MK , Paquet C , Delhaye S , Shin Y *et al* (2013) Rev‐erb‐alpha modulates skeletal muscle oxidative capacity by regulating mitochondrial biogenesis and autophagy. Nat Med 19: 1039–1046 23852339 10.1038/nm.3213 PMC3737409

[emmm201809390-bib-0056] Woods A , Dickerson K , Heath R , Hong SP , Momcilovic M , Johnstone SR , Carlson M , Carling D (2005) Ca^2+^/calmodulin‐dependent protein kinase kinase‐beta acts upstream of AMP‐activated protein kinase in mammalian cells. Cell Metab 2: 21–33 16054096 10.1016/j.cmet.2005.06.005

[emmm201809390-bib-0057] Wu J , Ruas JL , Estall JL , Rasbach KA , Choi JH , Ye L , Bostrom P , Tyra HM , Crawford RW , Campbell KP *et al* (2011) The unfolded protein response mediates adaptation to exercise in skeletal muscle through a PGC‐1alpha/ATF6alpha complex. Cell Metab 13: 160–169 21284983 10.1016/j.cmet.2011.01.003 PMC3057411

[emmm201809390-bib-0058] Xiao B , Sanders MJ , Underwood E , Heath R , Mayer FV , Carmena D , Jing C , Walker PA , Eccleston JF , Haire LF *et al* (2011) Structure of mammalian AMPK and its regulation by ADP. Nature 472: 230–233 21399626 10.1038/nature09932 PMC3078618

[emmm201809390-bib-0059] Yamamoto K , Amako M , Yamamoto Y , Tsuchihara T , Nukada H , Yoshihara Y , Arino H , Fujita M , Uenoyama M , Tachibana S *et al* (2013) Therapeutic effect of exendin‐4, a long‐acting analogue of glucagon‐like peptide‐1 receptor agonist, on nerve regeneration after the crush nerve injury. Biomed Res Int 2013: 315848 23984340 10.1155/2013/315848 PMC3747455

[emmm201809390-bib-0060] Zhang X , Zuo X , Yang B , Li Z , Xue Y , Zhou Y , Huang J , Zhao X , Zhou J , Yan Y *et al* (2014) MicroRNA directly enhances mitochondrial translation during muscle differentiation. Cell 158: 607–619 25083871 10.1016/j.cell.2014.05.047 PMC4119298

[emmm201809390-bib-0061] Zhang H , Ryu D , Wu Y , Gariani K , Wang X , Luan P , D'Amico D , Ropelle ER , Lutolf MP , Aebersold R *et al* (2016) NAD(+) repletion improves mitochondrial and stem cell function and enhances life span in mice. Science 352: 1436–1443 27127236 10.1126/science.aaf2693

[emmm201809390-bib-0062] Zheng H , Whitman SA , Wu W , Wondrak GT , Wong PK , Fang D , Zhang DD (2011) Therapeutic potential of Nrf2 activators in streptozotocin‐induced diabetic nephropathy. Diabetes 60: 3055–3066 22025779 10.2337/db11-0807 PMC3198067

[emmm201809390-bib-0063] Zou MH , Hou XY , Shi CM , Kirkpatick S , Liu F , Goldman MH , Cohen RA (2003) Activation of 5′‐AMP‐activated kinase is mediated through c‐Src and phosphoinositide 3‐kinase activity during hypoxia‐reoxygenation of bovine aortic endothelial cells. Role of peroxynitrite. J Biol Chem 278: 34003–34010 12824177 10.1074/jbc.M300215200

