# Supplementary material for: Mitochondrial glycerol 3‐phosphate dehydrogenase promotes skeletal muscle regeneration
Source: EMBO Mol Med. 2018 Nov 2;10(12):e9390. doi: 10.15252/emmm.201809390 (PMC6284384; doi:10.15252/emmm.201809390)
Supplement: Supplementary file 1 — Appendix [file EMMM-10-e9390-s001.pdf]

Appendix

Mitochondrial glycerol 3-phosphate dehydrogenase promotes skeletal muscle regeneration

Xiufei Liu[1,†], M.D.; Hua Qu[1,†], M.D., Ph.D.; Yi Zheng[1,†], Ph.D.; Qian Liao[1,†], M.D.; Linlin Zhang[1], M.D.; Xiaoyu Liao[1], Ph.D.; Xin Xiong[1], M.D.; Yuren Wang[1], M.D.; Rui Zhang[1], M.D.; Hui Wang[1], M.D., Ph.D.; Qiang Tong[1], M.D.; Zhenqi Liu[2], M.D.; Hui Dong[3], Ph.D.; Gangyi Yang[4], M.D., Ph.D.; Zhiming Zhu[5], M.D., Ph.D.; Jing Xu[1], M.D.; and Hongting Zheng[1,*], M.D., Ph.D.

Table of contents

Appendix Figures

Appendix Figure S1. mGPDH does not affect glucose uptake and insulin signaling in differentiated C2C12 myoblasts.

Appendix Figure S2. The expression characteristic of mGPDH during muscle regeneration.

Appendix Figure S3. mGPDH does not affect the satellite cell marker PAX7 and the satellite cell activation marker MyoD.

Appendix Figure S4. Mdx mice exhibit increased mGPDH and myogenin basal expression.

Appendix Figure S5. The efficiency of AAV-mGPDH to target different muscles.

Appendix Figure S6. mGPDH does not affect cell cycle, apoptosis, autophagy or IGF-1.

Appendix Figure S7. mGPDH does not significantly affect mitochondrial encoded OXPHOS genes.

Appendix Figure S8. mGPDH regulates AMPK independent of LKB1.

Appendix Figure S9. The effect of cGPDH on skeletal muscle regeneration.

Appendix Tables

Appendix Table 1. Clinical characteristics of obese patients and normal subjects.

Appendix Table 2. List of primers and siRNA sequences

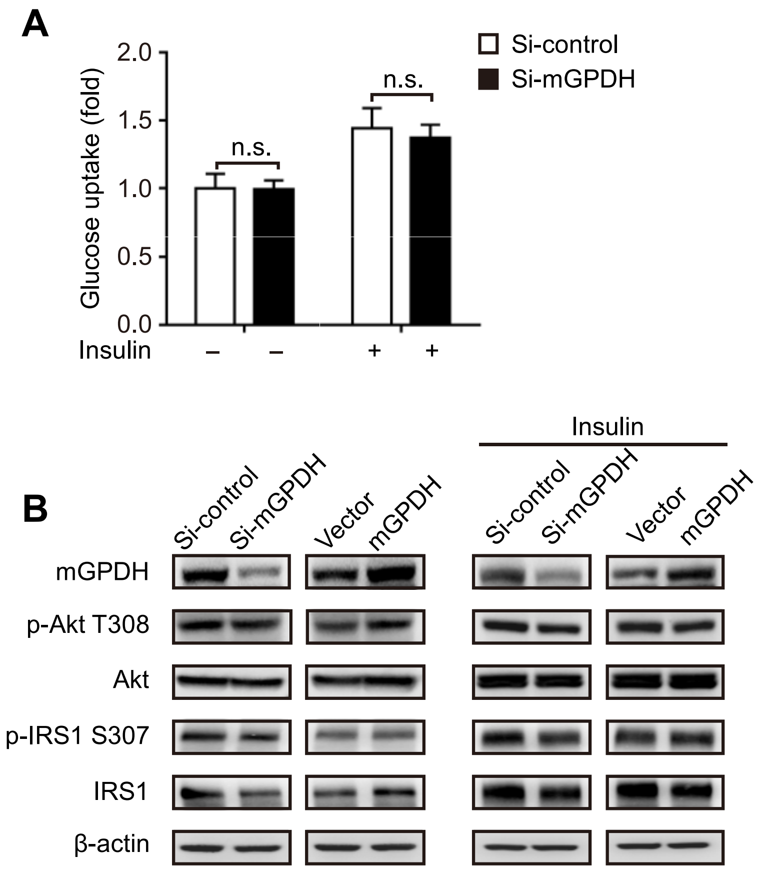

Appendix Fig S1. mGPDH does not affect glucose uptake and insulin signaling in differentiated C2C12 myoblasts. A,B C2C12 myocytes were induced to differentiation for 4 days and were subsequently transfected with mGPDH siRNA or plasmid, glucose uptake (A) and immunoblots of Akt and insulin receptor substrate 1 (IRS1) (B) with or without insulin stimulation (100 nM) were measured.

Data information: Data are presented as the mean ± s.e.m. $n = 3$ for all panels. n.s., not significant. Unpaired t-test was used for all panels.

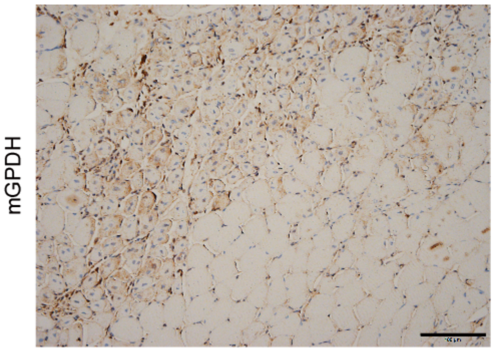

Appendix Figure S2. The expression characteristic of mGPDH during muscle regeneration.
Immunostaining of mGPDH in the GA muscles from C57BL/6J mice at day 7 post CTX intramuscular injection.

Data information: Data are presented as the mean ± s.e.m. Scale bars represent 100 μm.

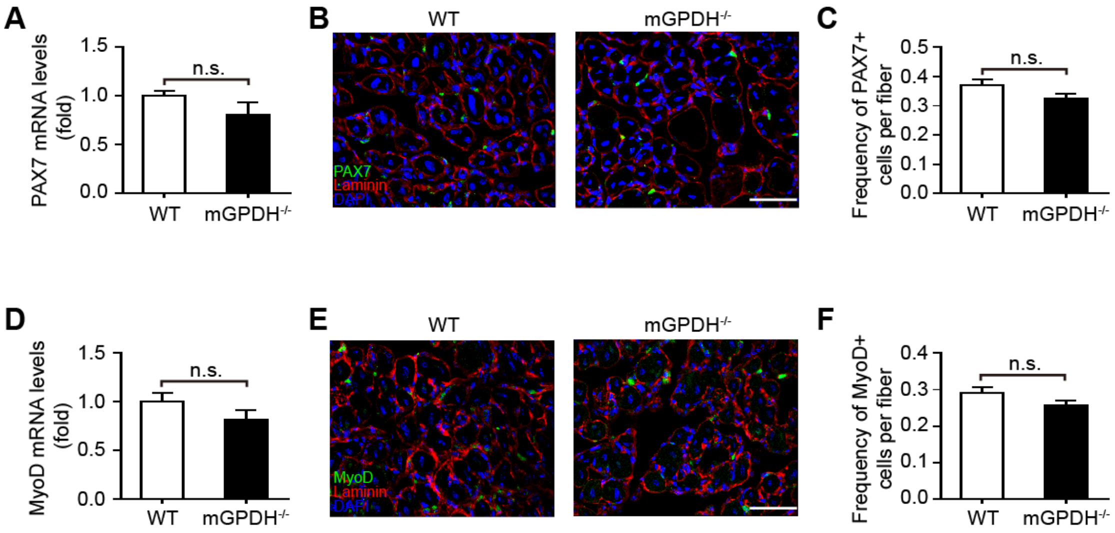

Appendix Fig S3. mGPDH does not affect the satellite cell marker PAX7 and the satellite cell activation marker MyoD.

A-F qRT-PCR of paired box protein 7 (PAX7) (A) and myoblast determination protein (MyoD) (D), immunofluorescence staining of PAX7 (green, B) and MyoD (green, E) and their corresponding quantifications (C and F) in GA muscles from WT and mGPDH$^{-/-}$ mice at day 7 post CTX injury.

Data information: Data are presented as the mean ± s.e.m. Scale bars represent 50 μm in panels B and E. In panels A-F, *n* = 6 mice per group; in panels B, C, E and F, 3 sections were obtained per mouse. n.s. not significant. Unpaired *t*-test was used for all panels.

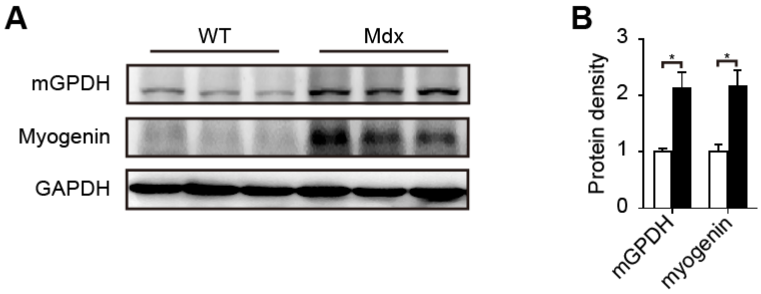

Appendix Figure S4. Mdx mice exhibit increased mGPDH and myogenin basal expression.

A,B Immunoblots of mGPDH and myogenin in GA muscle of 12-week-old WT and mdx mice (A). Quantifications represent mGPDH and myogenin protein levels (B).

Data information: Data are presented as the mean ± s.e.m. In panels A and B, *n* = 3 mice per group. **P* < 0.05. Unpaired *t*-test was used for panel B.

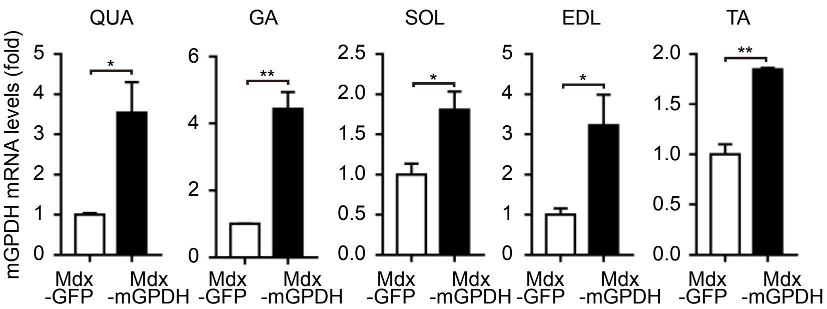

Appendix Figure S5. The efficiency of AAV-mGPDH to target different muscles.

Mdx mice were treated with AAV-mGPDH via tail vein, and after 4 weeks, mGPDH levels in indicated muscles were analyzed by qRT-PCR.

Data information: Data are presented as the mean ± s.e.m. *P < 0.05, **P < 0.01. Unpaired *t*-test was used for all panels.

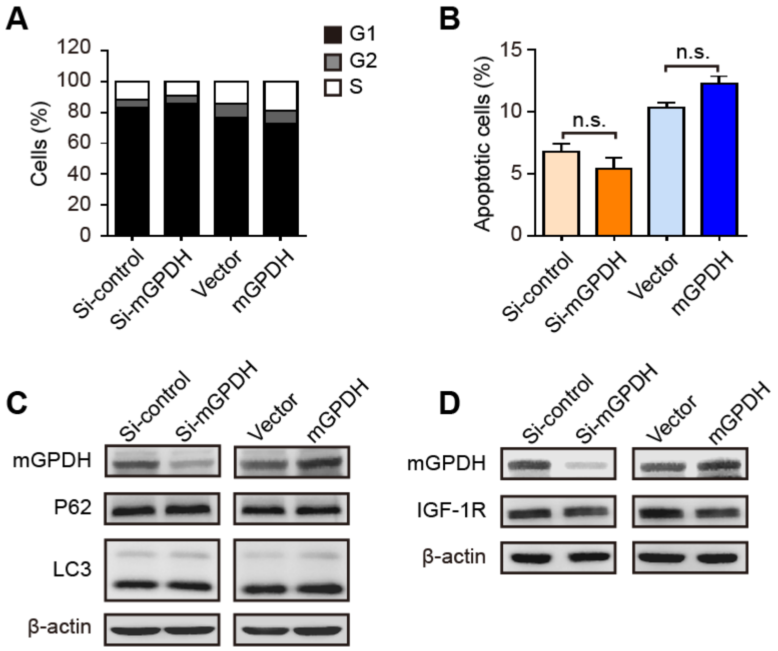

Appendix Figure S6. mGPDH does not affect cell cycle, apoptosis, autophagy or IGF-1.

A-D, C2C12 myocytes were transfected with mGPDH siRNA or plasmid for 24 h. Cells were then incubated in differentiation medium for an additional 24 h, and the cell cycle (A), and apoptosis (B), and immunoblots of p62, LC3 (C) and insulin-like growth factor-1 receptor (IGF-1R) (D) were assessed.

Data information: Data are presented as the mean ± s.e.m. In panels A-D, *n* = 3. n.s. not significant. Unpaired *t*-test was used for all panels.

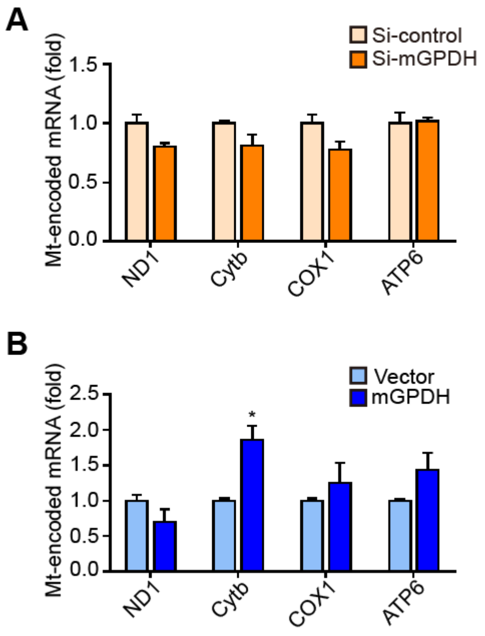

Appendix Figure S7. mGPDH does not significantly affect mitochondrial encoded OXPHOS genes.

A,B C2C12 myocytes were transfected with mGPDH siRNA (A) or plasmid (B) for 24 h, cells were then incubated in differentiated medium for an additional 24 h, and mRNA levels of indicated mitochondrial encoded oxidative phosphorylation (OXPHOS) genes were assessed.

Data information: Data are presented as the mean ± s.e.m. In panels A and B, $n = 3$. *$P < 0.05$. Unpaired *t*-test was used for all panels.

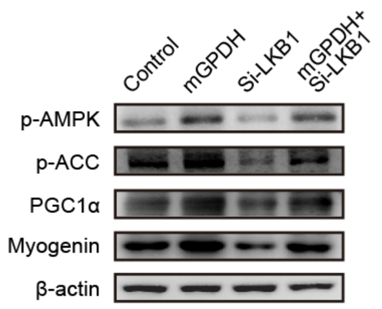

Appendix Figure S8. mGPDH regulates AMPK independent of LKB1.

C2C12 myocytes were transfected with LKB1 siRNA for 24 h and overexpressed with the mGPDH plasmid for 24 h and cells were then incubated in differentiated medium for an additional 24 h. Western blots were performed with the indicated antibodies.

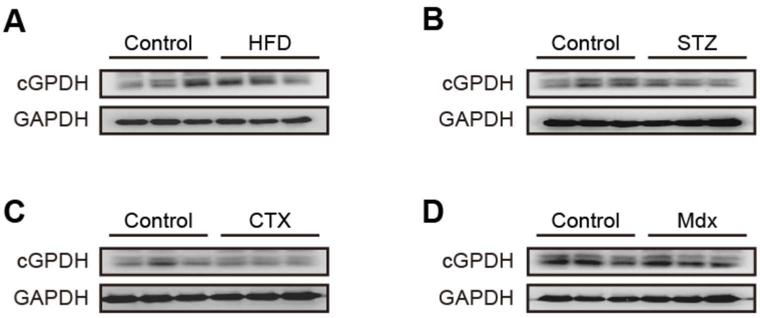

Appendix Fig S9. The effect of cGPDH on skeletal muscle regeneration.
A-D Western blot analysis of cGPDH in GA muscles of HFD (A), STZ (B), CTX (C) and mdx (D) mouse models.

Data information: n = 3 mice per group.

Appendix Table S1. Clinical characteristics of obese patients and normal subjects.

Characteristics	Control	Obese
**N**	18	11
**Age (year)**	43.33±3.80	43.73 ±3.24
**Body composition**		
Weight (kg)	58.72±1.47	76.55 ±2.25***
BMI (kg/m^2)	21.69 ±0.35	27.32 ±0.32***
**Laboratory parameters**		
ALT (U/L)	40.52±6.27	42.19±9.76
AST (U/L)	34.66±4.55	40.59±9.21
GGT (U/L)	57.23±17.89	85.50±24.56
Cr (μmol/L)	34.27±7.52	48.60±10.97

Data are presented as the mean ± s.e.m. ***$P < 0.001$, by Student's *t*-test. BMI, body mass index; ALT, alanine transaminase; AST, aspartate transaminase; GGT, glutamyl transferase; Cr, creatinine.

Appendix Table S2. List of primers and siRNA sequences

**Primers**		
mGPDH	Forward	GTGCGCAAAATGGATGATA
	Reverse	CATCCCCTCTTCTCACTTCAA
Myogenin	Forward	TACGTCCATCGTGGACAGCAT
	Reverse	TCAGCTAAATTCCCTCGCTGG
MyHC	Forward	AGCGAATCGAGGCCCAGA
	Reverse	TGTCGTACTTGGGAGGGTTCA
PAX7	Forward	GAGTTCGATTAGCCGAGTGC
	Reverse	CGGGTTCTGATTCCACATCT
MyoD	Forward	ACATAGACTTGACAGGCCCCGA
	Reverse	AGACCTTCGATGTAGCGGATGG
Myh3	Forward	ACCTCTAGCCGGATGGT
	Reverse	AATTGTCAGGAGCCACGAAAAT
Utrophin	Forward	ATCTTGTCGGGCTTTCCAC
	Reverse	ATCCAAAGGCTTTCCCAGAT
GAPDH	Forward	TGAACGGGAAGCTCACTG
	Reverse	TCCACCACCCTGTTGCTG
COX2	Forward	ATAACCGAGTCGTTCTGCCAAT
	Reverse	TTTCAGAGCATTGGCCATAGAA
NDUFS8	Forward	TACGTCCATCGTGGACAGCAT
	Reverse	TCAGCTAAATTCCCTCGCTGG
SDHb	Forward	ACCCCTTCTCTGTCTACCG
	Reverse	AATGCTCGCTTCTCCTTGTAG
Uqcrc1	Forward	ATCAAGGCACTGTCCAAGG
	Reverse	TCATTTTCCTGCATCTCCCG
COX5b	Forward	ACCCTAATCTAGTCCCGTCC
	Reverse	CAGCCAAAACCAGATGACAG
ATP5a1	Forward	CATTGGTGATGGTATTGCGC
	Reverse	TCCCAAACACGACAACTCC

ND1	Forward	TGCACCTACCCTATCACTCA
	Reverse	GGCTCATCCTGATCATAGAATGG
Ctyb	Forward	CCCACCCCATATTAAACCCG
	Reverse	GAGGTATGAAGGAAAGGTATAAG
COX1	Forward	CCCAGATATAGCATTCCCACG
	Reverse	ACTGTTCATCCTGTTCCTGC
ATP6	Forward	TCCCAATCGTTGTAGCCATC
	Reverse	TGTTGGAAAGAATGGAGTCGG
18S	Forward	AGGGGTTCGGGATTTGTG
	Reverse	GACCAGGCGGAACAGAGA
cGPDH	Forward	GAAGGCAAAAAGCTGACTGAGAT
	Reverse	CTTGTGCTGGAGGATGCTGTAT
IL-1 β	Forward	CCAGGATGAGGACATGAGCA
	Reverse	CGGAGCCTGTAGTGCAGTTG
TNF- α	Forward	ACTGGCAGAAGAGGCACTCC
	Reverse	GCCACAAGCAGGAATGAGAA
IL-6	Forward	TCCATCCAGTTGCCTTCTTG
	Reverse	AAGCCTCCGACTTGTGAAGTG
Myh7	Forward	CAGGTCTGGCTCTGAGCATT
	Reverse	ATCATCCAGGAAGCGTAGCG
Myh2	Forward	TGCCAGTAAGGGTCTGTGAG
	Reverse	GCGCTTTTTGCCTCGATAGG
Myh4	Forward	GGCAAACAAGCATTTACACAAC
	Reverse	ATCCGTCTCATATTTCGTCCTC
Myl4	Forward	TCTTTGACAAAGAAAGCAACGG
	Reverse	CAGACATGATGTGCTTGACAAA
Myh8	Forward	GAGGACCAAATATGAGACCGAT
	Reverse	TCCTCTCCACATCAATCATGAG
**siRNA sequences**		
mGPDH-siRNA	GAGAUGACUUAAACUCUAATT	
LKB1-siRNA	GGAGAAGCAGAAGATGTAT	

116
