# Supplementary material for: Mitochondrial glycerol 3‐phosphate dehydrogenase promotes skeletal muscle regeneration
Source: EMBO Mol Med. 2018 Nov 2;10(12):e9390. doi: 10.15252/emmm.201809390 (PMC6284384; doi:10.15252/emmm.201809390)
Supplement: Supplementary file 2 — Expanded View Figures PDF [file EMMM-10-e9390-s002.pdf]

Expanded View Figures

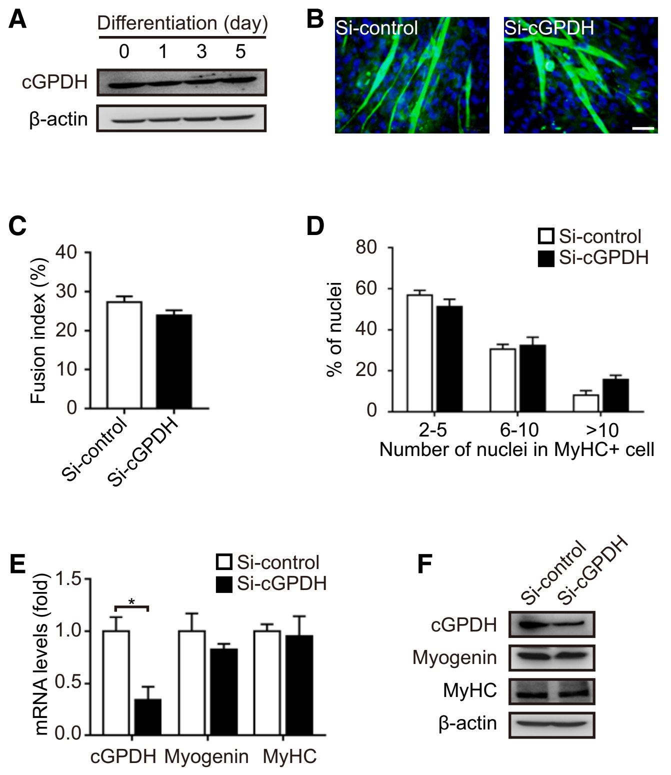

Figure EV1. Effect of cGPDH on myoblast differentiation.

A cGPDH expression during C2C12 myocyte differentiation.

B–D Representative images of MyHC immunofluorescence (B) of C2C12 myocytes transfected with siRNA targeting cGPDH; the fusion index (C) and the distribution of nuclei per myotube (D) were calculated.

E, F qRT–PCR (E) and Western blot analysis (F) of myogenin and MyHC in C2C12 myocytes transfected with siRNA targeting cGPDH.

Data information: Data are presented as the mean ± s.e.m. Scale bars represent 50 μm in panel (B). In panels (A–F), $n = 3$. *$P < 0.05$. Unpaired t-test was used for all analyses except in panel (D), where the Kolmogorov–Smirnov test was used.

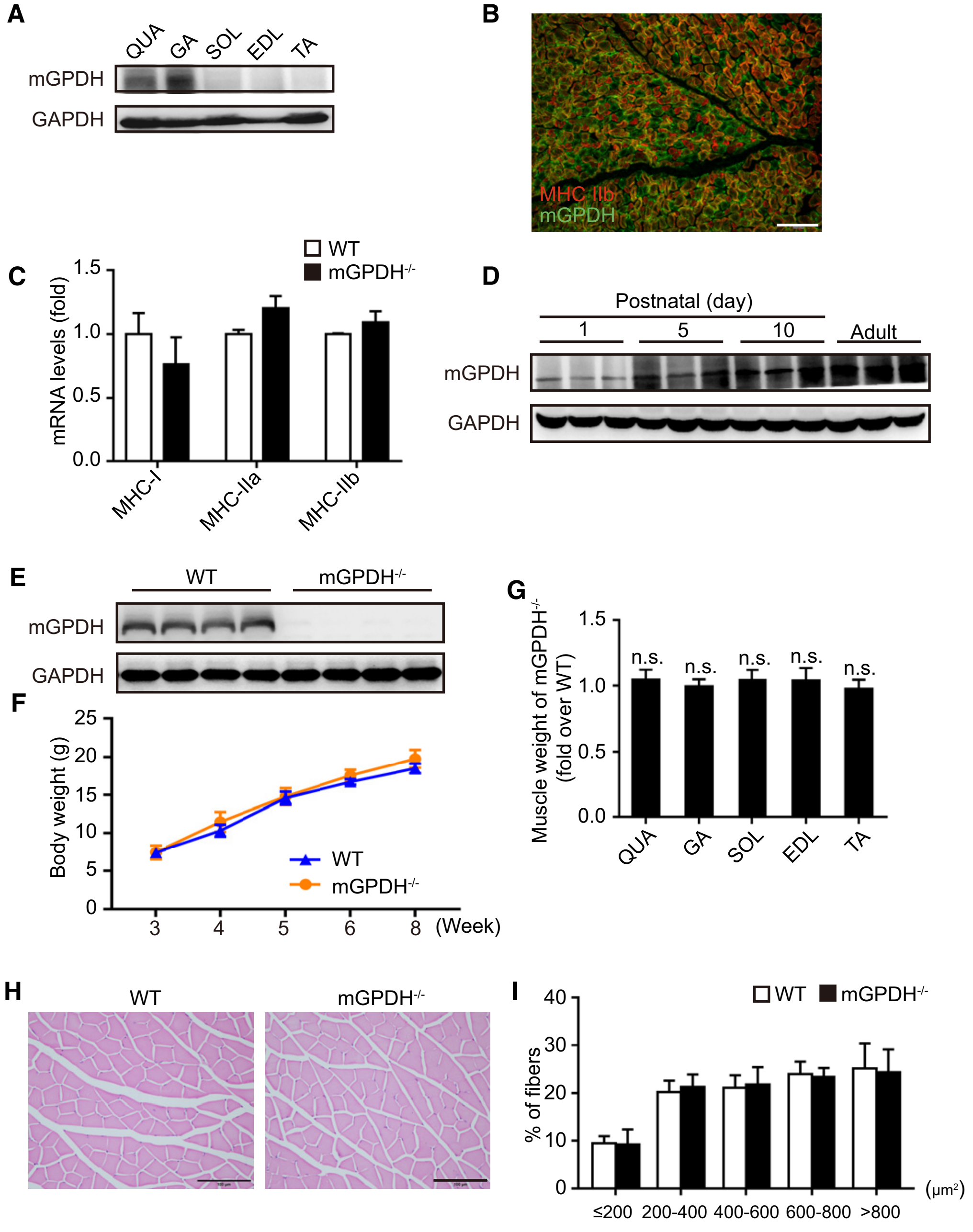

Figure EV2. mGPDH is not essential to muscle development.

A Immunoblot of mGPDH in the quadriceps (QUA), gastrocnemius (GA), soleus (SOL), extensor digitorum longus (EDL), and tibialis anterior (TA) muscles of 8-week-old C57BL/6J mice.

B Immunofluorescence showing localization of mGPDH with fiber type marker MHC IIb on cryosections from uninjured GA muscle of 8-week-old C57BL/6J mice.

C qRT–PCR analyses of the indicated fiber type markers (MHC I, IIa, and IIb) in the uninjured GA muscles of 8-week-old WT and mGPDH$^{-/-}$ mice.

D Immunoblot of mGPDH in C57BL/6J mouse skeletal muscle at postnatal days 1, 5, and 10 and 8 weeks.

E Immunoblot of mGPDH in the GA muscle of 8-week WT and mGPDH$^{-/-}$ mice.

F Body weight of WT and mGPDH$^{-/-}$ mice at the indicated week of age.

G Muscle weight of the indicated 8-week-old mGPDH$^{-/-}$ mice normalized to WT.

H, I Hematoxylin–eosin (H&E) staining (H) and average myofiber cross-sectional area (CSA) (I) in the GA muscle of 8-week-old WT and mGPDH$^{-/-}$ mice.

Data information: Data are presented as the mean ± s.e.m. Scale bars represent 200 μm in panel (B) and 100 μm in panel (H). In panels (A and D), n = 3 mice per group; in panels (B and C), n = 6 mice per group; in panels (E–I), n = 4 mice per group; in panels (H and I), three sections were obtained per mouse. n.s., not significant. Unpaired t-test was used for all analyses except in panel (I), where the Kolmogorov–Smirnov test was used.

© 2018 The Authors

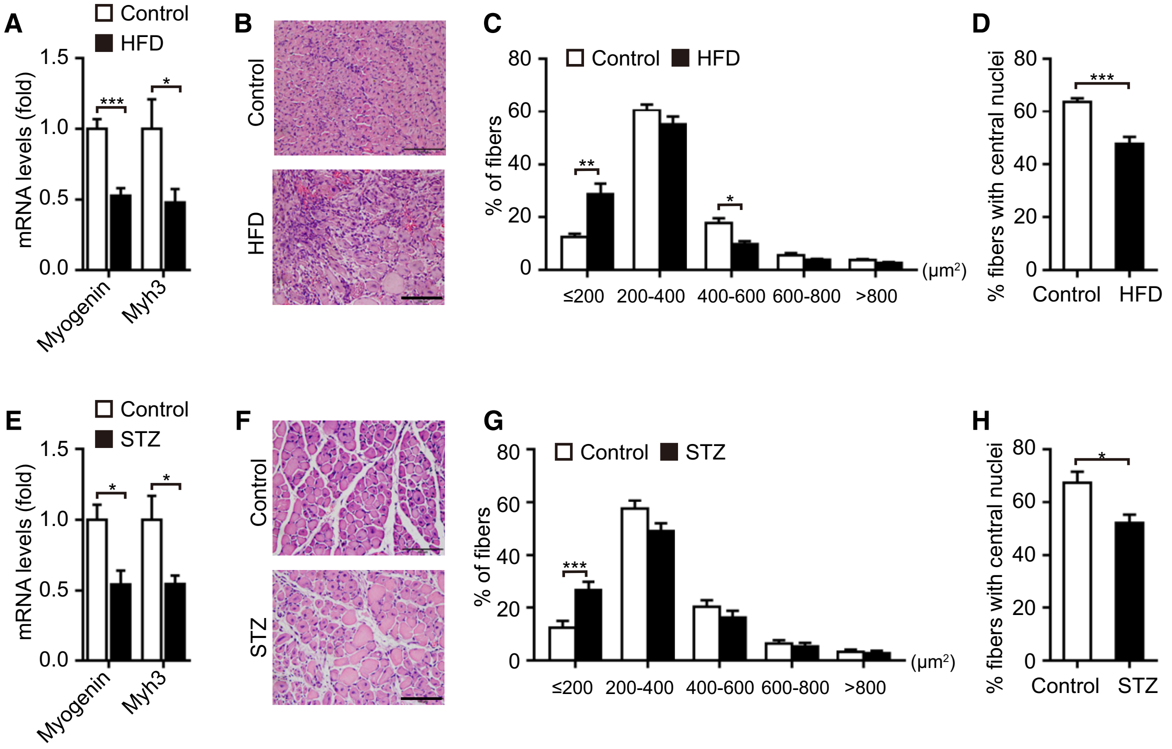

Figure EV3. Skeletal muscle regeneration is impaired in HFD-fed mice and STZ-injected mice.

A–D Gastrocnemius (GA) muscles were obtained from HFD-fed mice at day 7 post-CTX injury. Quantification of myogenin and myh3 by qRT–PCR (A), representative images of H&E staining (B), distribution of the CSA (C), and percentage of myofibers with central nuclei (D).

E–H GA muscles were obtained from STZ-treated mice 4 weeks after STZ injection and at day 7 post-CTX injury. Quantification of myogenin and myh3 by qRT–PCR (E), representative images of H&E staining (F), distribution of the fibers CSA (G), and percentage of myofibers with central nuclei (H).

Data information: Data are presented as the mean ± s.e.m. Scale bars represent 100 μm in panels (B and F). $n = 6$ mice per group. *$P < 0.05$, **$P < 0.01$, ***$P < 0.001$. Unpaired *t*-test was used for all analyses except in panels (C and G), where the Kolmogorov–Smirnov test was used.

© 2018 The Authors

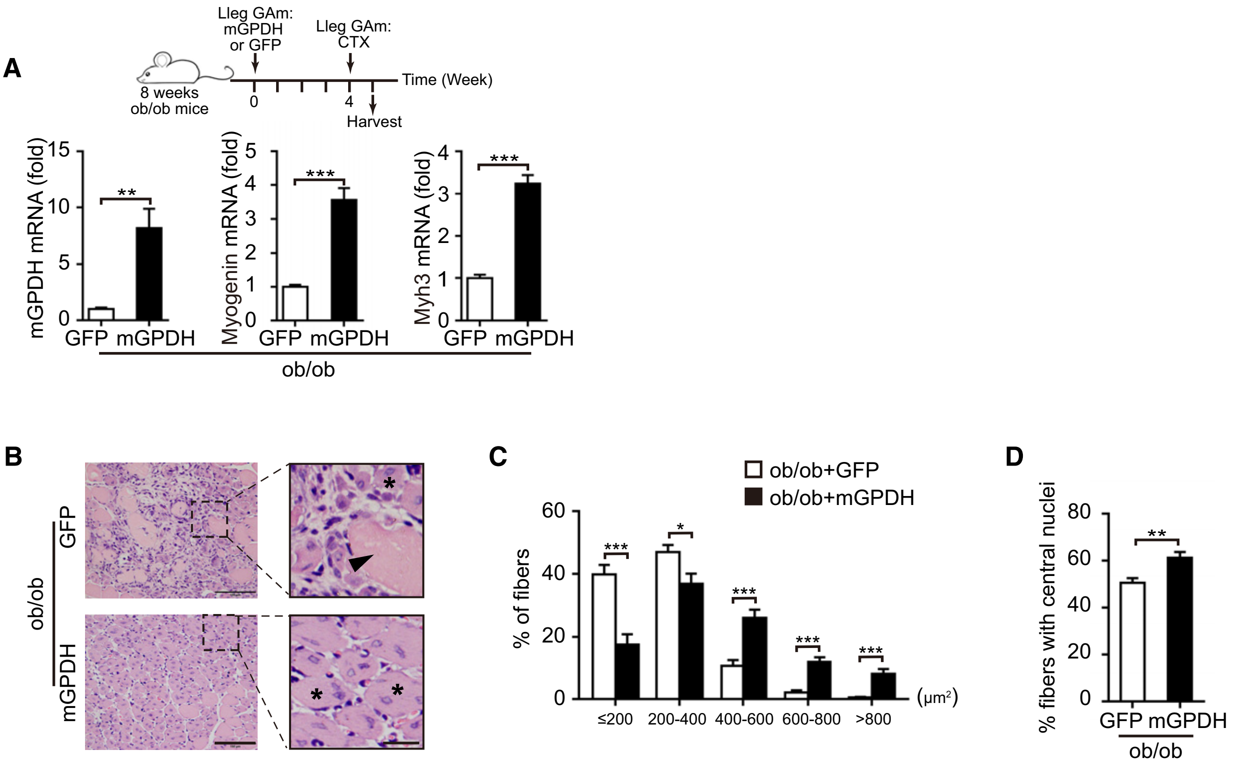

Figure EV4. mGPDH activation improves skeletal muscle regeneration in *ob/ob* mice.

A–D Experimental setup (A, upper panel) and qRT–PCR of mGPDH, myogenin, and myh3 gene expressions (A, bottom panel), H&E staining (arrowhead, necrotic myofibers; asterisks, regenerating fibers) (B), distribution of CSA (C), and percentage of fibers with central nuclei (D) in GA muscle from AAV-mGPDH-treated *ob/ob* mice at day 7 post-CTX.

Data information: Data are presented as the mean ± s.e.m. Scale bars represent 100 μm (25 μm for magnification insets) in panel (B). In panels (A–D), $n = 6$ mice per group; in panels (B–D), three sections were obtained per mouse. *$P < 0.05$, **$P < 0.01$, ***$P < 0.001$. Unpaired *t*-test was used for all analyses except in panel (C), where the Kolmogorov–Smirnov test was used.

© 2018 The Authors

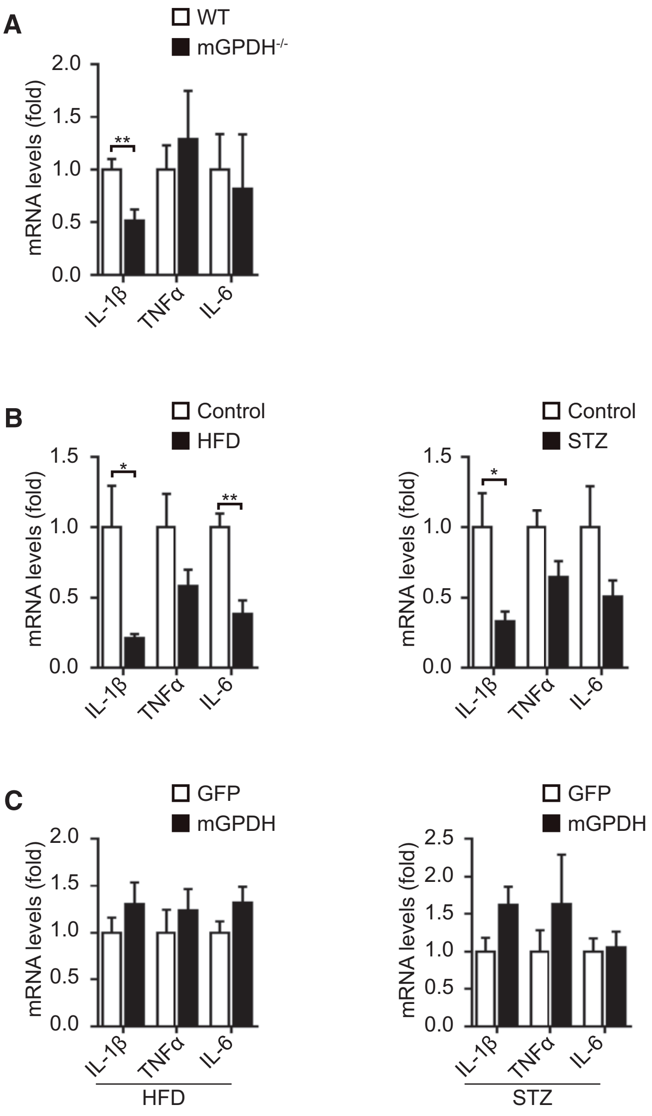

Figure EV5. Effect of mGPDH on inflammatory signaling.

A–C Quantification of the indicated inflammatory cytokines by qRT–PCR in GA muscles of mGPDH$^{-/-}$ mice (A), HFD-fed and STZ-treated mice (B), and HFD-fed and STZ-treated mice intramuscularly injected with AAV-mGPDH (C) at day 7 post-CTX injury.

Data information: Data are presented as the mean ± s.e.m. *n* = 6 mice per group. *$P < 0.05$, **$P < 0.01$. Unpaired *t*-test was used for all panels.

© 2018 The Authors
