# Supplementary figures and images for: Mitochondrial glycerol 3‐phosphate dehydrogenase promotes skeletal muscle regeneration
Source: EMBO Mol Med. 2018 Nov 2;10(12):e9390. doi: 10.15252/emmm.201809390 (PMC6284384; doi:10.15252/emmm.201809390)

**Figure 1**

**Panel B**

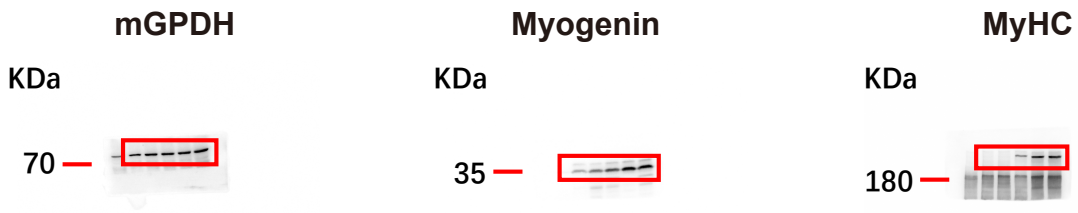

**Panel C**

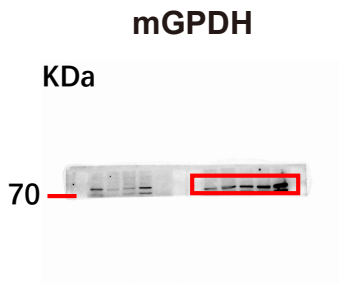

**Panel H**

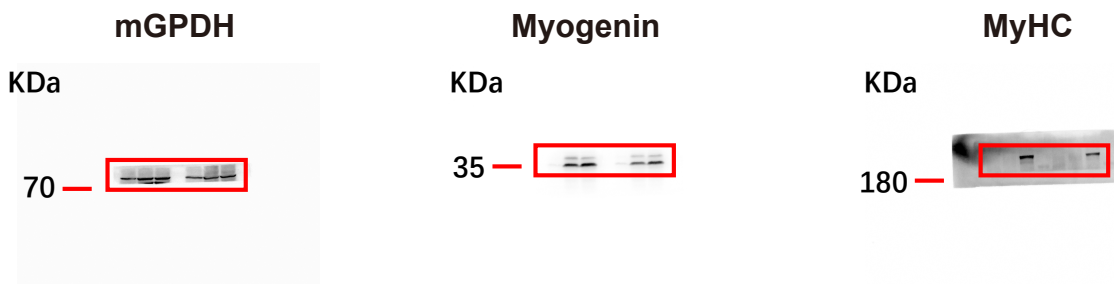

Supplement: Supplementary file 5 — Source Data for Figure 1 [file EMMM-10-e9390-s004.pdf]

**Figure 2**

**Panel B**

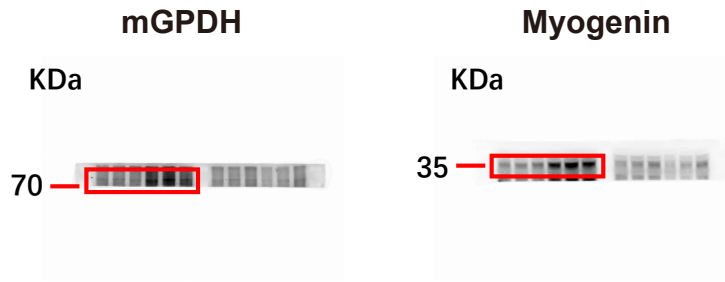

**Panel K**

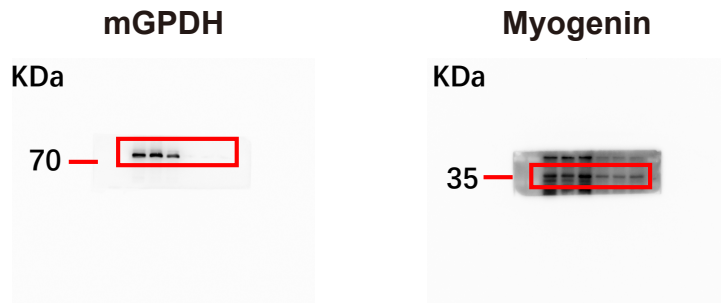

Supplement: Supplementary file 6 — Source Data for Figure 2 [file EMMM-10-e9390-s005.pdf]

Figure 4

Panel A

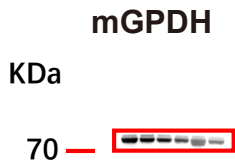

Panel C

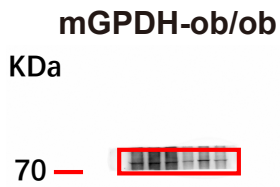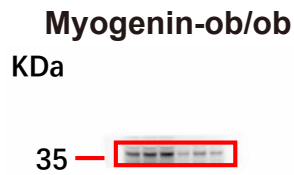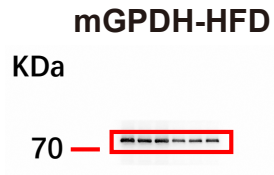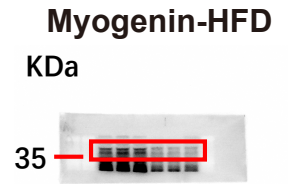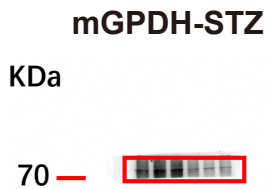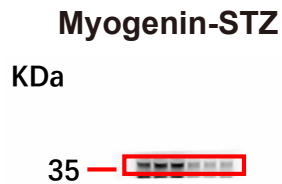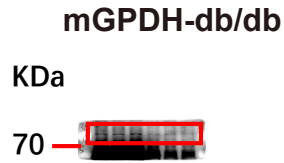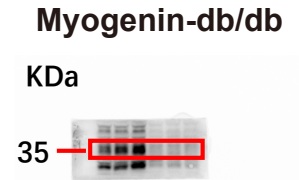

Panel N

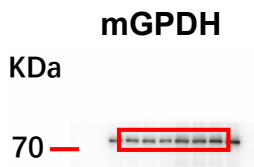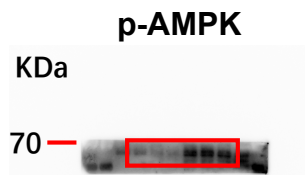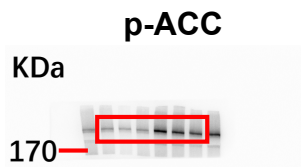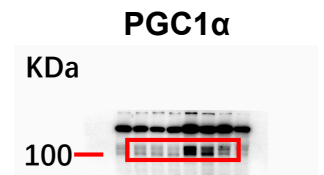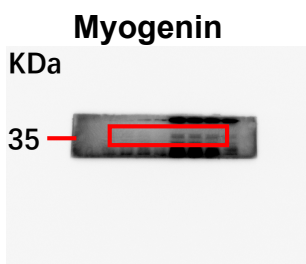

Supplement: Supplementary file 8 — Source Data for Figure 4 [file EMMM-10-e9390-s007.pdf]
