# Supplementary material for: Mitochondrial glycerol 3‐phosphate dehydrogenase promotes skeletal muscle regeneration
Source: EMBO Mol Med. 2018 Nov 2;10(12):e9390. doi: 10.15252/emmm.201809390 (PMC6284384; doi:10.15252/emmm.201809390)

Figure 3

Panel D

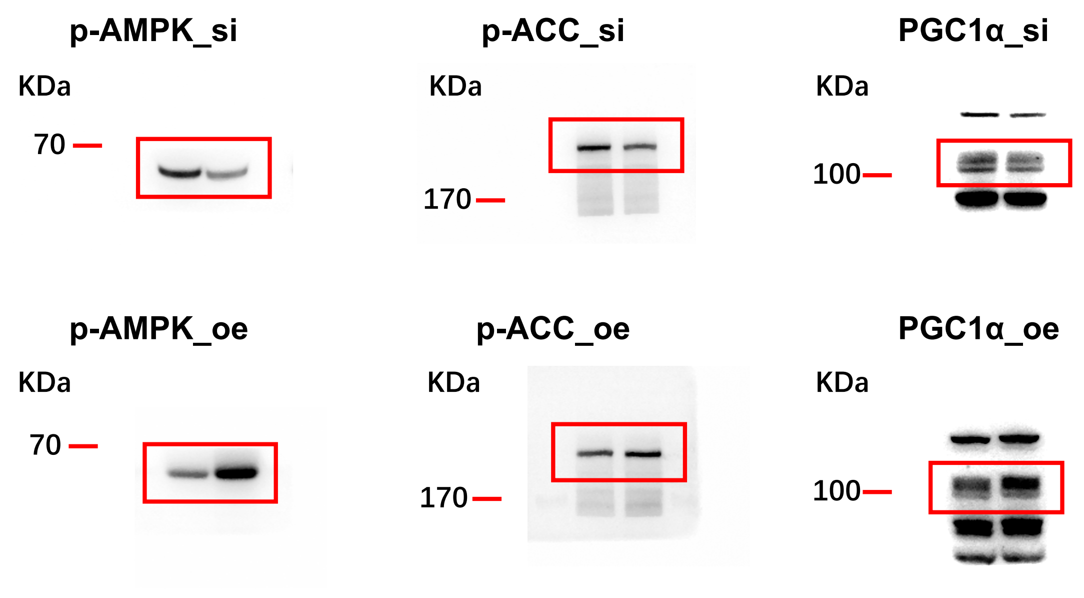

Panel G

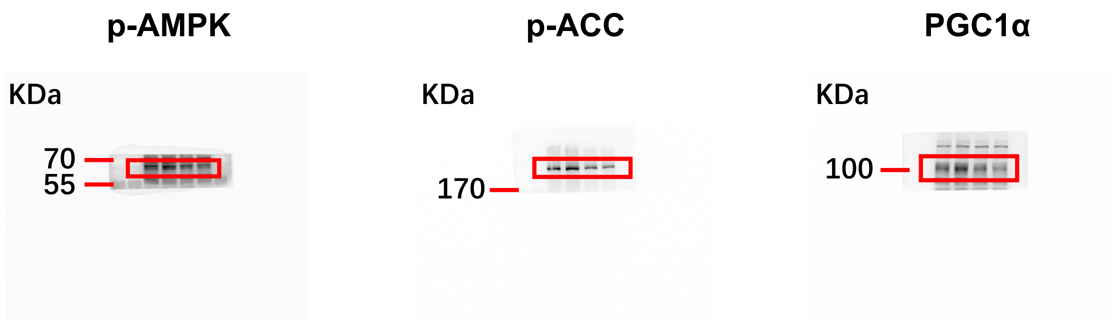

Panel K

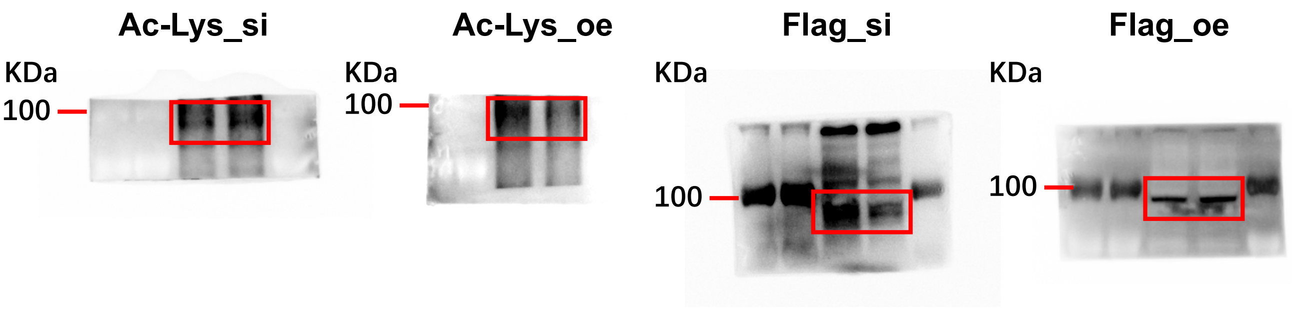

Panel L

C-myc

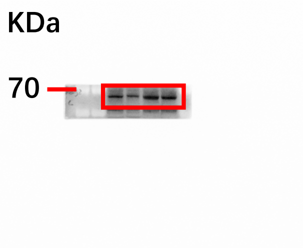

Myogenin

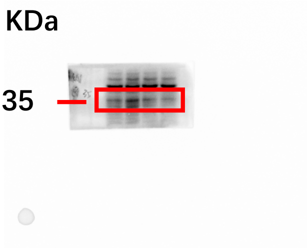

Panel Q

p-AMPK

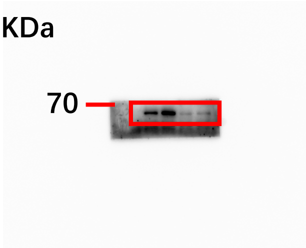

p-ACC

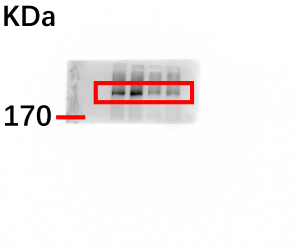

PGC1α

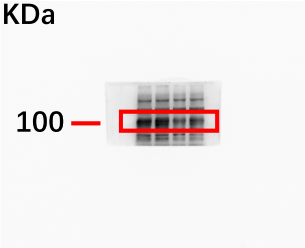

Myogenin

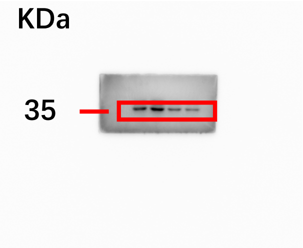

Panel R

p-AMPK

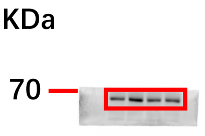

p-ACC

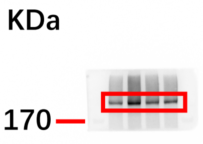

Supplement: Supplementary file 7 — Source Data for Figure 3 [file EMMM-10-e9390-s006.pdf]
